# PANTHER Score: Protein-Affinity for Nucleic Target-binding, Hybridization, and Energy Regression

**DOI:** 10.1261/rna.080646.125

**Published:** 2026-02

**Authors:** Parisa Aletayeb, Akash Deep Biswas, Stefano Rocca, Carmine Talarico, Giulio Vistoli, Alessandro Pedretti

**Affiliations:** 1 Dipartimento di Scienze Farmaceutiche, Università degli Studi di Milano, Milano 20133, Italy; 2 EXSCALATE, Dompé Farmaceutici S.p.A., Napoli 80131, Italy

**Keywords:** protein-RNA interactions, binding free energy (Δ*G*), pairwise interaction energies, machine learning models, predictive modeling, RNA-therapeutics

## Abstract

Although protein–RNA interactions are crucial for many biological processes, predicting their binding free energies (Δ*G*) is a challenging task due to limited available experimental data and the complexity of these interactions. To address this issue, we developed a machine learning–based model designed to predict energy-based scores for protein–RNA complexes, called PANTHER Score. By applying a local-to-global approach, we proposed a methodology further subdivided into five steps: (1) We derived 87,117 pairwise local interaction energies from 331,744 MD-derived interactions across 46 curated protein–RNA complexes; (2) we trained ML models on pairwise interaction features to predict local interaction energies without performing MD simulations; (3) we integrated predicted local interaction energies using a local-to-global methodology, to compute model-specific PANTHER Score; (4) we evaluate model-specific PANTHER Score on an independent test set of seven complexes; and (5) we validated and selected the optimal model using an external stress set of 110 complexes with experimental Δ*G* values for implementation in the PANTHER Scoring pipeline. Among the regression models developed, Random Forest Regression exhibited the highest predictive performance as a model-specific PANTHER Score, achieveing a Pearson correlation (*r*) of 0.80 and MAE of 1.79 kcal/mol on the test set. It maintained strong predictive capabilities on the stress set (*r* = 0.64, MAE = 1.63 kcal/mol). Benchmarking against existing tools on the stress test set, the PANTHER Score demonstrated superior accuracy and reliability. This study highlights the effectiveness of MD and machine learning in addressing data limitations through innovative strategies, positioning the PANTHER Score as a robust tool for predicting protein–RNA binding affinities in biomolecular research, drug discovery and mainly in RNA-therapeutics.

## INTRODUCTION

Protein–RNA interactions play a central role in numerous key biological processes (Jones 2016), from gene expression to cellular signaling and regulation. Experimentally determined binding affinities provide critical insights into functions and specificity and can be used to better evaluate protein–nucleic acid interactions. Binding affinity quantitatively describes how strongly a protein interacts with nucleic acid and is essential for regulating processes like transcription, replication, and repair. Often, a high binding affinity correlates with effective gene regulation and cellular function (Lunde et al. 2007; Harini et al. 2023). Understanding binding affinities also provides mechanistic insights by elucidating the recognition mechanisms between proteins and nucleic acids and allowing a more precise understanding of the structural or sequence elements that can affect interactions (Yang and Deng 2020; Harini et al. 2023).

Experimental methods for determining the binding affinity of protein–nucleic acid complexes include several techniques, such as fluorescence spectroscopy as reported by Vivian and Callis in 2001, surface plasmon resonance as discussed by Katsamba in 2002, electrophoretic mobility shift assay as described by Hellman and Fried in 2007 and later by Ryder et al. in 2008, isothermal titration calorimetry as highlighted by Feig in 2009, and filter binding assay as proposed by Rio in 2012. While being accurate, these methods are both costly and labor-intensive. This restricts their applicability in high-throughput screenings of large libraries as the field of drug discovery evolves to focus on increasingly larger biomolecules such as RNA-aptamers (Bell et al. 2020). As the demand for reliable techniques to investigate protein–nucleic acid interactions has rapidly grown, computational methods have gained popularity to overcome these limitations (Ding and Kihara 2018; Yang and Deng 2020; Bheemireddy et al. 2022; Baek et al. 2024).

Emerging computational methodologies have led to the widespread utilization of molecular docking software for predicting binding conformations and ranking them through scoring functions (Li et al. 2010; Pons et al. 2011). Focusing on protein–RNA binding free energy, four notable protein–RNA web-based/standalone predictive tools are available: PRA-Pred (Harini et al. 2024), PRdeltaGPred (Hong et al. 2023), the structure-based model as proposed by Nithin et al. in 2019, and PredPRBA (Deng et al. 2019). PRdeltaGPred is a standalone program that utilizes complex structural features, including noninteraction surfaces, desolvation energy, hydrogen bond energy, and salt bridge energy, to predict experimental binding free energy. Similarly, PRA-Pred is a web-based tool that predicts Δ*G* value based on simple features such as base parameters, interaction energies, number of contacts, and hydrogen bonds. Next, Nithin et al. developed a structure-based model that predicts the Δ*G* value by considering interface parameters such as hydrophobicity, contact surface, and hydration pattern. Finally, PredPRBA (Deng et al. 2019) includes different predictive models depending on the interacting RNA structure.

At present, the limited availability of tools capable of accurately calculating the experimental Δ*G* value for protein–RNA interactions highlights a critical gap in computational biology. While considering the above-mentioned tools, the field urgently requires more robust, accessible, and accurate tools. Developing new computational-based methods to predict experimental binding free energy (BFE) will significantly enhance protein–RNA research, advancing our understanding of their roles in biological systems and supporting drug discovery efforts.

To address the need for specifically tailored approaches, we developed a machine learning (ML) model that predicts local energies between interacting amino acid–nucleotide bases of a protein–RNA complex, and later we integrate these local energy contributions through a scoring function, by a local-to-global approach. We name the whole process as Protein-Affinity for Nucleic Target-binding, Hybridization, and Energy Regression score (PANTHER Score), which reveals an encouraging correlation with the binding free energy between proteins and nucleic acids. Our approach focuses on local pairwise interactions between amino acids and nucleotide bases and can be subdivided into three parts: (i) A representative set of local interaction energies was derived from molecular dynamics (MD) simulations involving a training set composed by 46 curated protein–RNA complexes (train set 1 and train set 2); (ii) ML models were trained to predict these local interaction energies without performing MD runs but based on simple pairwise interaction features, such as amino acid type, nucleotide base type, the number of hydrogen bonds, and distances between interacting amino acids and nucleotide bases; and (iii) the so-predicted local energies were integrated by applying a local-to-global approach to calculate the model-specific PANTHER Score. The ML models and the resulting model-specific PANTHER Scores were initially tested and refined on a reduced test set composed of seven protein–RNA complexes with experimental Δ*G* values. Optimal performance was achieved through the application of Random Forest Regression (RFR). The resulting RFR model-specific PANTHER Score demonstrated a correlation coefficient (*r*) of 0.80 and a mean absolute error of 1.63 kcal/mol. Furthermore, when tested on a markedly more extended nonredundant protein–RNA data set of 110 complexes, the RFR model-specific PANTHER Score achieved an encouraging (*r*) of 0.64. These results underscore the potential of ML models for accurately predicting pairwise interaction energies and the reliability of the local-to-global approach in evaluating the protein–RNA binding energies.

To further validate our model, we compared its performance on a data set of 110 uncorrelated protein–RNA complexes with existing techniques. In detail, the comparison involved the PredPRBA and PRA-Pred, which are easily accessible through web service and the obtained correlations between experimental and predicted Δ*G* values gave (*r*) equal to 0.64, 0.18, and 0.18 for PANTHER Score, PredPRBA, and PRA-Pred, respectively. This comparison underscores the potential of our model for evaluating protein–RNA interactions and demonstrates its robustness and superior accuracy when compared to currently available methods.

## RESULTS AND DISCUSSION

### Evaluation of local-to-global scoring methodology

The proposed method is based on the reliability of the local-to-global approach, which implies an additive nature of the Δ*G* values where an interaction score (kcal/mol) can be computed by integrating the contributions of the local interaction energies of amino acid–nucleotide bases, which we here refer to as MD-derived local-to-global score. These local contributions can be obtained either from MD simulations or predicted by ML models. Hence, the preliminary but crucial step of our study is assessing the reliability of this fragmental approach, at least when integrating the local energies as derived from MD runs.

**FIGURE 1.**
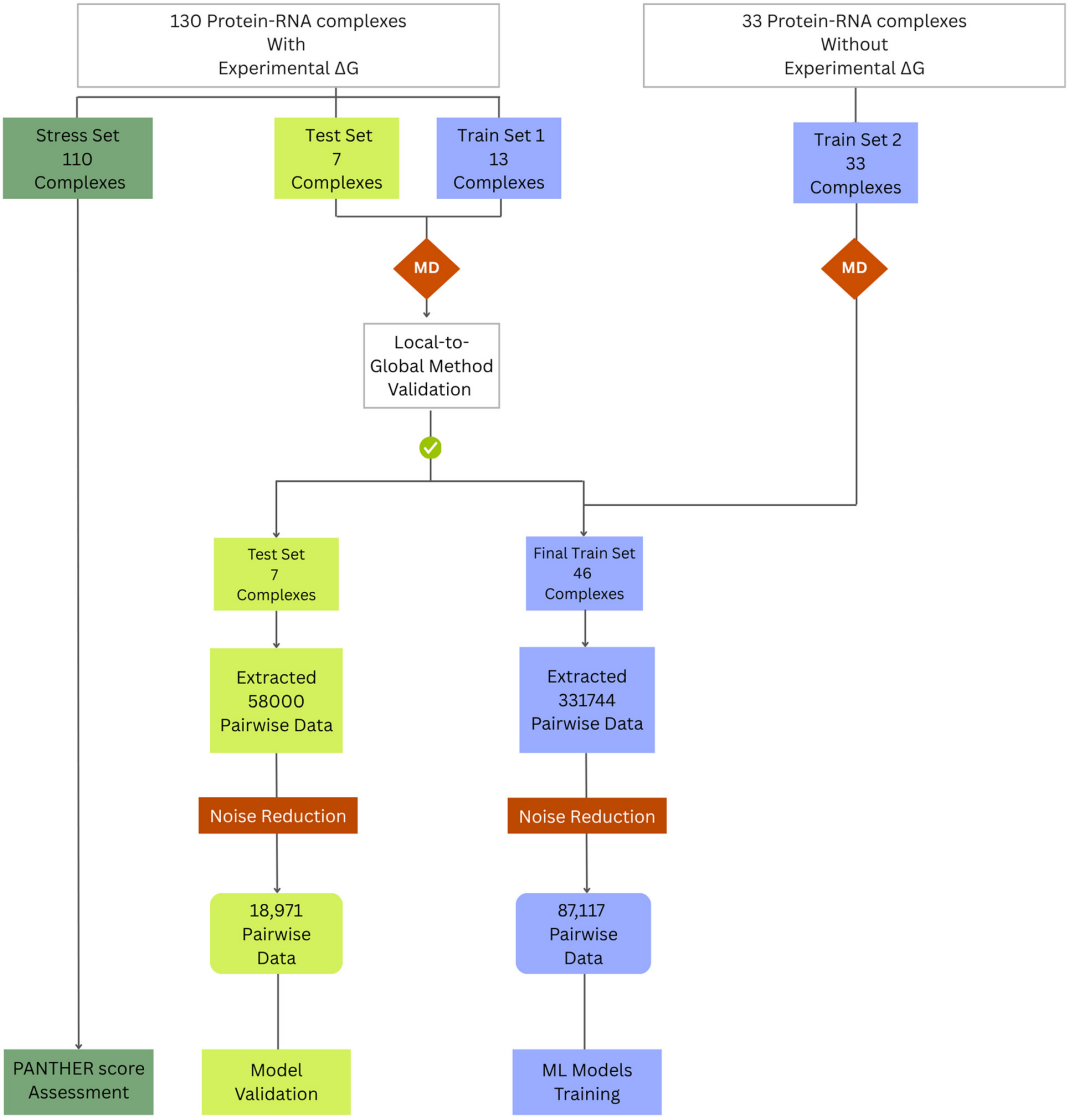
Schematic representation of the data set preparation and validation workflow. Protein–RNA complexes with and without experimental Δ*G* values were subjected to MD simulations, followed by the local-to-global energy strategy to compute the MD-derived PANTHER Scores. The validated scores were used to construct the final training and test sets for ML model development and validation.

**FIGURE 2.**
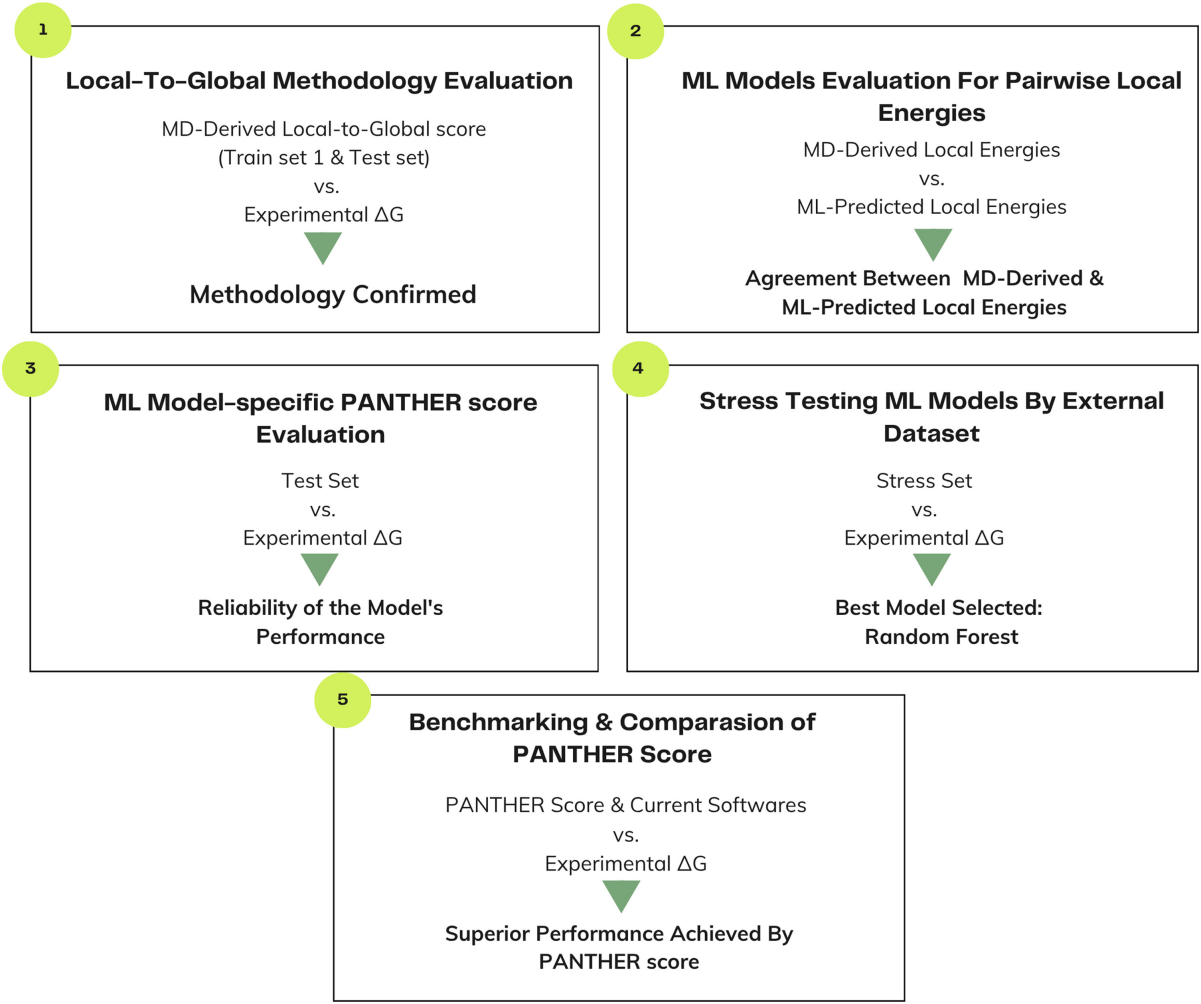
Step-by-step workflow followed for developing the PANTHER Score is explained through this schematic workflow.

As the MD-derived local energies will later serve as training data for various ML models, it is essential to apply an initial evaluation to ensure the local-to-global methodology exhibits a robust correlation with experimental data. Accordingly, out of the 130 complexes with experimental Δ*G*, initially we kept away 110 complexes for the stress set, and out of remaining 20 complexes, 13 complexes were considered for training set 1 and seven complexes for test set (refer to Fig. 1; Supplemental Tables S1, S2). The MD simulation for both training set 1 and test set was carried out, and the local interaction energy between amino acid and nucleotide for each complex was extracted. Subsequently, the local interactions energies were integrated to the local-to-global score (methodology described in subsection “Local-to-global integration”), and the local-to-global scores were correlated with the experimental Δ*G* values of the simulated complexes. The complete workflow is shown in Figure 2.

**FIGURE 3.**
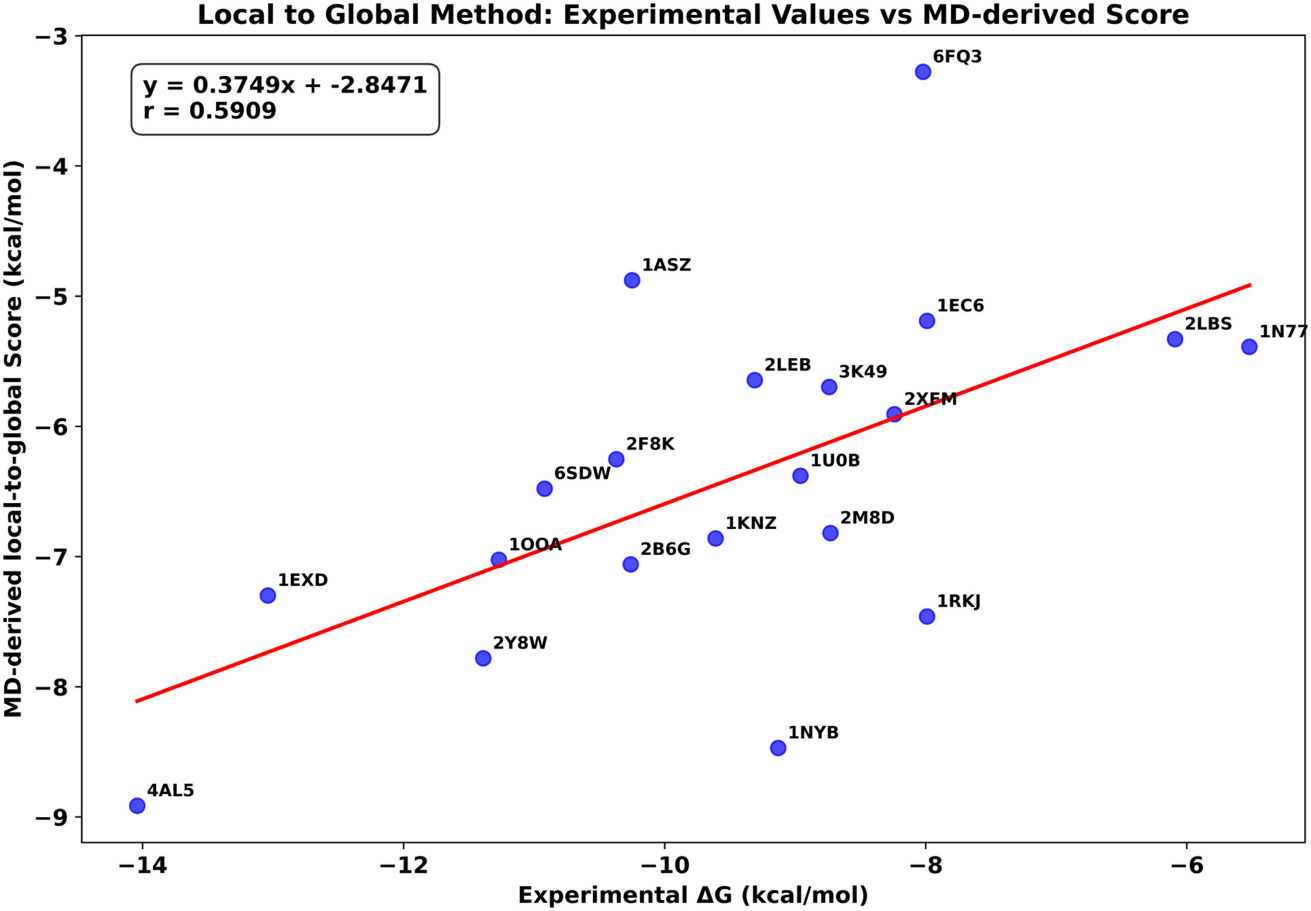
Correlation between the MD-derived local-to-global score and experimental Δ*G* values for 20 protein–RNA complexes (13 from train set 1 + seven from test set), showing a Pearson correlation coefficient (*r*) of 0.60, which confirms the reliability of our fragmental local-to-global approach.

The results obtained are shown in Figure 3, which plots MD-derived local-to-global score versus the experimental Δ*G* and reveals an encouraging correlation coefficient (*r*) of 0.60 obtained from 20 protein–RNA complexes. Furthermore, the plots of each train set 1 (*r* = 0.75) and test set (*r* = 0.59) are separately shown in Supplemental Figures S1 and S2, supporting evidence that the local-to-global scoring system can be considered reliable. As shown in Figure 3 and Supplemental Figures S1 and S2, the regression line slopes deviate significantly from the ideal 45° slope, indicating that while strong correlations exist, the predicted Δ*G* values do not precisely match experimental values. However, the primary goal of this study is to develop a scoring function that conveniently correlates with experimental binding affinities rather than to exactly predict absolute Δ*G* values.

Even though the primary objective of our study involves the development of an innovative protein–RNA binding affinity score which does not require MD simulations, the preliminary results seem to confirm the additive nature of binding energy, which can be calculated by integrating the contribution of the local interactions and suggest that such a local-to-global approach can be clearly exploited when using MD-derived local energies to obtain reliable and interpretable predictions of MD-derived PANTHER Score. This data-driven approach has the potential to drastically reduce computation time while maintaining satisfactory predictive accuracy. Together, this two-tiered strategy, starting with physics-based calculations and moving toward ML-based predictions, sets up a pipeline for a scalable and efficient framework to estimate binding affinities in protein–RNA systems.

After validating the reliability of our approach, we expanded the training set by including 33 additional protein–RNA complexes as train set 2 (refer to Fig. 1). At this stage, we could include complexes without experimental Δ*G* values because the method's capability had already been assessed. We performed MD simulation for all three sets: training sets 1 and 2 and test set. From the MD simulations, we extracted local interaction energies for each amino acid–nucleotide pair across all protein–RNA trajectories. This yielded 87,117 noise-free data points for the combined training set (set 1 + set 2) and 18,971 noise-free data points for the independent test set (Fig. 1). The Materials and Methods subsection “Extraction of pairwise local energies and noise reduction technique” details the noise reduction technique used in this study.

### ML models and performance on test set

#### Data sets preparation and analysis

The performance of any ML model is intrinsically linked to the quality of the data set used for training. To ensure robust predictions, we conducted a thorough structural analysis of each protein–RNA complex, validating the residues and bonds as outlined under the Materials and Methods (see Materials and Methods subsection “Data sets selection and preparation”). We then performed MD simulations on 53 complexes (train set 1, train set 2, and test set), from which we extracted MD-derived local energies between the interacting amino acid–nucleotide base (Fig. 1). From this initial pool of 53 complexes, seven structures were designated as the test set, thereby defining curated training (refer to Supplemental Table S1 for the list) and test data sets (refer to Supplemental Table S2 for the list).

**FIGURE 4.**
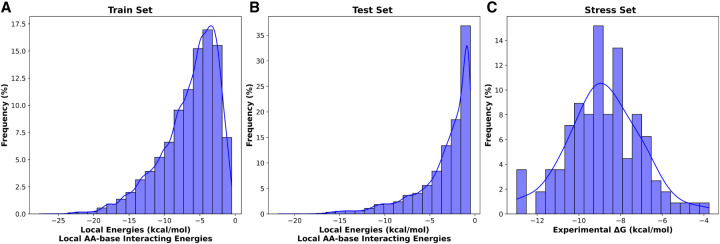
Distribution of MD-derived local energies of the interacting amino acid–nucleotide bases for the (2A) training set and (2B) test set, alongside the experimental Δ*G* for the stress set (2C). The distributions illustrate consistency and variation across the monitored energy values, with a fitted line highlighting the trends.

Furthermore, as detailed in the Materials and Methods subsection “Development of prediction models,” we trained ML models to predict the local energies derived from MD trajectories using simple descriptors, such as amino acid residues, nucleotide bases, local interaction energies, the number of hydrogen bonds formed, and interatomic distances. To optimize predictive performances, we tried a broad range of regression algorithms, beginning with linear regression as a baseline and extending to more sophisticated algorithms capable of handling complex data. These included RFR, Extreme Gradient Boosting (XGBoost), Gradient Boosting (GBoost), Stacking Regression, and Neural Networks. Moreover, we studied the diversity of local interaction energies as input data in the training set, which is illustrated in Figure 4A, showing a wide distribution ranging from weak to strong interactions. To ensure that the ML models can accurately predict local energies across diverse interaction strengths, we evaluated them on a test set containing MD-derived local energies with an equally broad distribution (Fig. 4B). This wide range challenges the models to generalize across both weak and strong local interactions. The similarity between training and test set distributions (Fig. 4A,B) indicates that model evaluation is unbiased.

In contrast, Figure 4C reports the distribution of the experimental Δ*G* values for the 110 complexes, which exhibits a symmetric, approximately Gaussian distribution with fewer extreme values. In this study, these experimental binding affinities serve as an important validation benchmark for overall model performance and to guide the selection process of the optimal ML model for PANTHER Score prediction. This validation step is crucial to confirm that integration of local energetic contributions accurately reproduces a score of binding affinity, which achieves high correlation with the experimental Δ*G* values. The predictive performance of the models is evaluated in two stages: first on the test set (Table 1, detailed in the “Model evaluation” subsection) and then on the larger stress data set (“ML model assessment” subsection).

**TABLE 1.** Performance metrics of various machine learning models on the test set for PANTHER Score predictions

Model	Test set			
	*r*	MAE	*r*_*s*_	ρ-value
Random Forest Regression	0.80	1.79	0.77	0.04
GBoosting Regression	0.79	1.71	0.77	0.04
Neural Network	0.68	1.59	0.77	0.04
Stacked Ensemble	0.77	1.68	0.77	0.04
XGBoosting Regression	0.72	1.63	0.77	0.04
Linear Regression	0.69	1.62	0.52	0.22

Metrics include Pearson correlation coefficient (*r*), mean absolute error (MAE), Spearman's rank correlation coefficient (*r*_*s*_), and ρ-value.

#### Agreement between MD- and ML-derived local energies

Having established the distribution of MD-derived local energies used as input, the next step is to determine whether ML models trained on MD-derived descriptors can correctly predict the local energy values. Only if the ML models can successfully recover the MD signal at the local level, the integration to perform local-to-global score will be reliable. Such an agreement was quantified by Pearson correlation coefficients (*r*) between MD-derived and ML-predicted local energies, as computed per complex and summarized across models (Supplemental Table S2). While showing expected variability, the analysis of the specific performances (see Supplemental Table S2) reveals rather satisfactory results with (*r*) almost always >0.5. Interestingly, the (*r*) mean value of each protein unravels a marked variability, suggesting that some complexes perform clearly better. These differences can be ascribed to the structural complexity, which is, in turn, related to the number of local energies to be predicted. Taken together, the high average correlation values across diverse protein systems and machine learning approaches (overall [*r*] mean = 0.72) indicate that the developed ML models can effectively capture the patterns in local energy as originally derived from computationally expensive MD simulations.

Considering only one exemplificative case, Supplemental Figure S3 shows a detailed visualization of the correlations between MD-derived and ML-derived local energy values for the 1EC6 protein system as obtained by six different ML approaches. The scatter plots demonstrate that all models capture the general trends in local energies, with points colored according to their distance from the trend line to highlight prediction accuracy. From Supplemental Table S2 we observe that from the ML-predicted local energies of 1EC6, we obtained (*r*) of 0.80, 0.82, 0.84, 0.82, 0.84, and 0.76 for RFR, GBoost, Neural Network, Stacked Ensemble, XGBoost, and Linear Regression, respectively. Overall, when the correlations are calculated for all seven PDB IDs from test set, their results are encouraging, with mean (*r*) typically ≥0.7, for most complexes.

Thus, the performance demonstrated by the ML models provides compelling evidence that machine learning can serve as a viable alternative to MD simulations for predicting the interacting local energies, provided that the training data set is of high quality and highly curated. All ML approaches tested show good correlations with MD data, confirming that the local energies of protein–RNA complexes can be successfully calculated by using these ML models. This finding is particularly significant given the computational resources typically required for MD simulations, while ML models might yield comparable results at a fraction of the computational cost and time. As the ML models can reliably reproduce the MD-derived local energies, we next assess how these models perform by comparing their predictions, integrated as model-specific PANTHER Scores, with the corresponding experimental Δ*G* values in the test set.

#### Model evaluation

As described under Materials and Methods (see “PANTHER Score” and “Local-to-global integration” sections), the PANTHER Score represents a descriptor of binding affinity for protein–RNA complexes. This score is derived by integrating ML-predicted local interaction energies, specifically those involving amino acid–nucleotide base pairs identified within a range of 9 Å distance from their center of mass. This integration of these local energetic contributions provides a measure of each complex's strength of binding affinity.

To ensure methodological accuracy, each ML model which includes RFR, Gradient Boosting, Neural Network, Stacked Ensemble, XGBoost, and Linear Regression was trained independently to predict local energies. As a result, each model produces its own prediction of the overall PANTHER Score for a given complex. Throughout this framework, these are referred to as model-specific PANTHER Scores until a particular ML model is finally selected as the optimal solution to predict the PANTHER Score (see Fig. 2 for workflow).

However, the predicted accuracy of the approach is assessed by comparing these model-specific PANTHER Scores to the experimental Δ*G* values. This comparison enables evaluation of each model's ability to predict binding affinities and guides the selection process for the optimal ML methodology within the framework.

**FIGURE 5.**
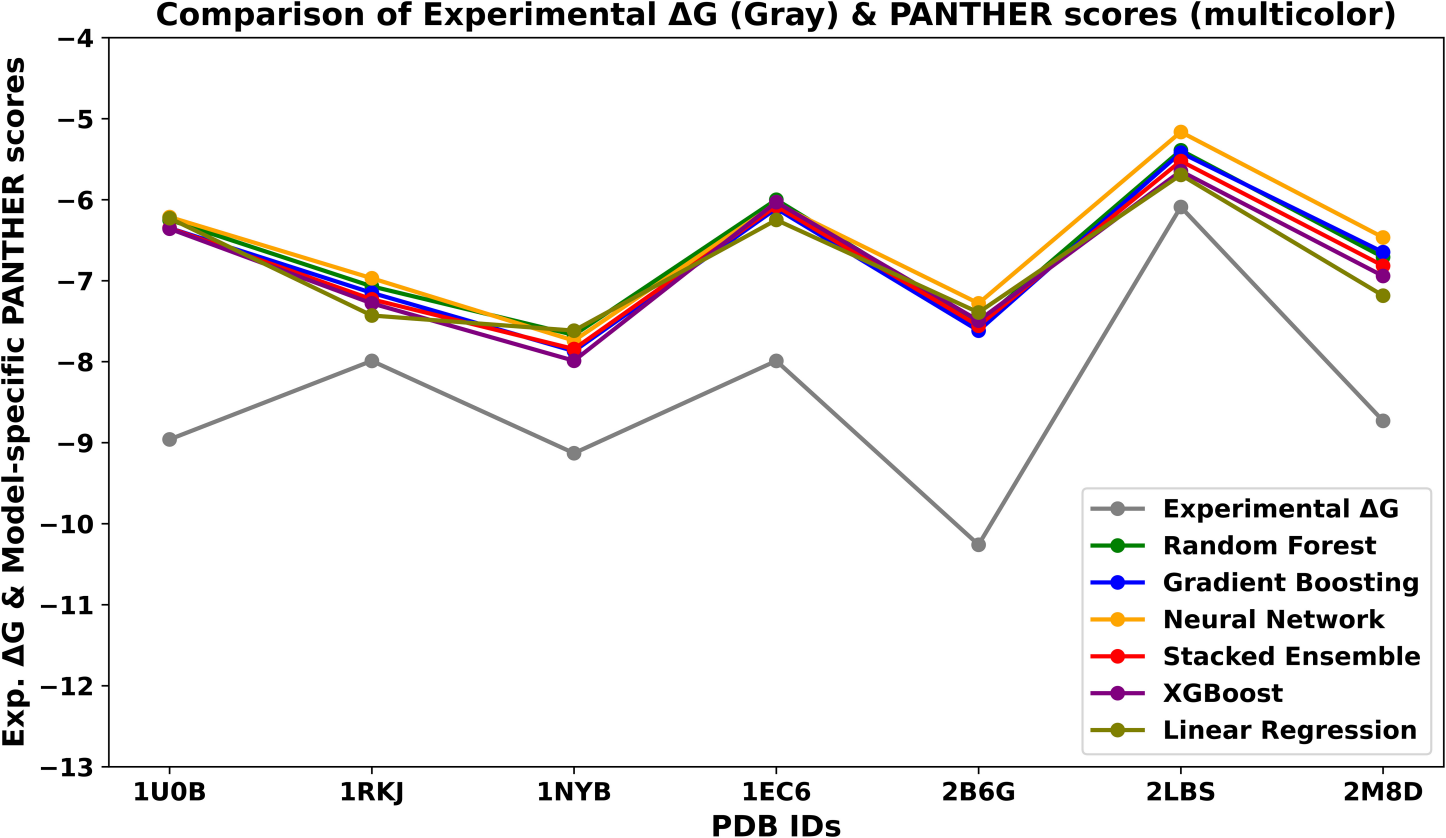
Comparison of experimental Δ*G* (kcal/mol) and model-specific PANTHER Scores predicted with various ML models for the test set. The *x*-axis represents the PDB IDs included in the test set, while the *y*-axis reports the corresponding experimental Δ*G* values (gray) and the model-specific PANTHER Scores predicted by various outputs (predicted local energies) of machine learning (ML) models conjugated with the here proposed local-to-global methodology. Model-specific PANTHER Scores obtained from Random Forest Regression, Gradient Boosting, Neural Network, Stacked Ensemble, XGBoost, and Linear Regression models are represented in green, blue, orange, red, magenta, and olive, respectively. This comparison was performed to evaluate the consistency and trends between experimental Δ*G* and PANTHER Scores predicted by various ML models. The results indicate no substantial deviation among the predictions of the different ML models, suggesting that no single model can be preferentially selected to define the final PANTHER Score. Therefore, all ML models were further evaluated using an unbiased external data set, referred to as the “Model evaluation,” as detailed in the Results and Discussion section.

As shown in Figure 5, the *x*-axis represents the PDB IDs of the test set complexes, while the *y*-axis shows the corresponding experimental Δ*G* values (gray line) and the PANTHER Scores predicted by the different ML models. The experimental Δ*G* values vary substantially (from approximately −6 to −12 kcal/mol), reflecting diverse binding affinities among the complexes. All ML-derived PANTHER Score predictions closely follow the experimental trend, exhibiting high consistency across models.

The correlation coefficients (*r* > 0.7; see Table 1) indicate good predictive performance, with only minor differences among models (Δ*r* < 0.15). The RFR and Gradient Boosting models achieve the highest correlations (*r* ≈ 0.8), although all ML models perform comparably. To further assess robustness, we also calculated additional statistical metrics, including the Spearman correlation coefficient (*r*_*s*_), mean absolute error (MAE), and ρ-values to evaluate statistical significance (data shown in Supplemental Table S3).

**FIGURE 6.**
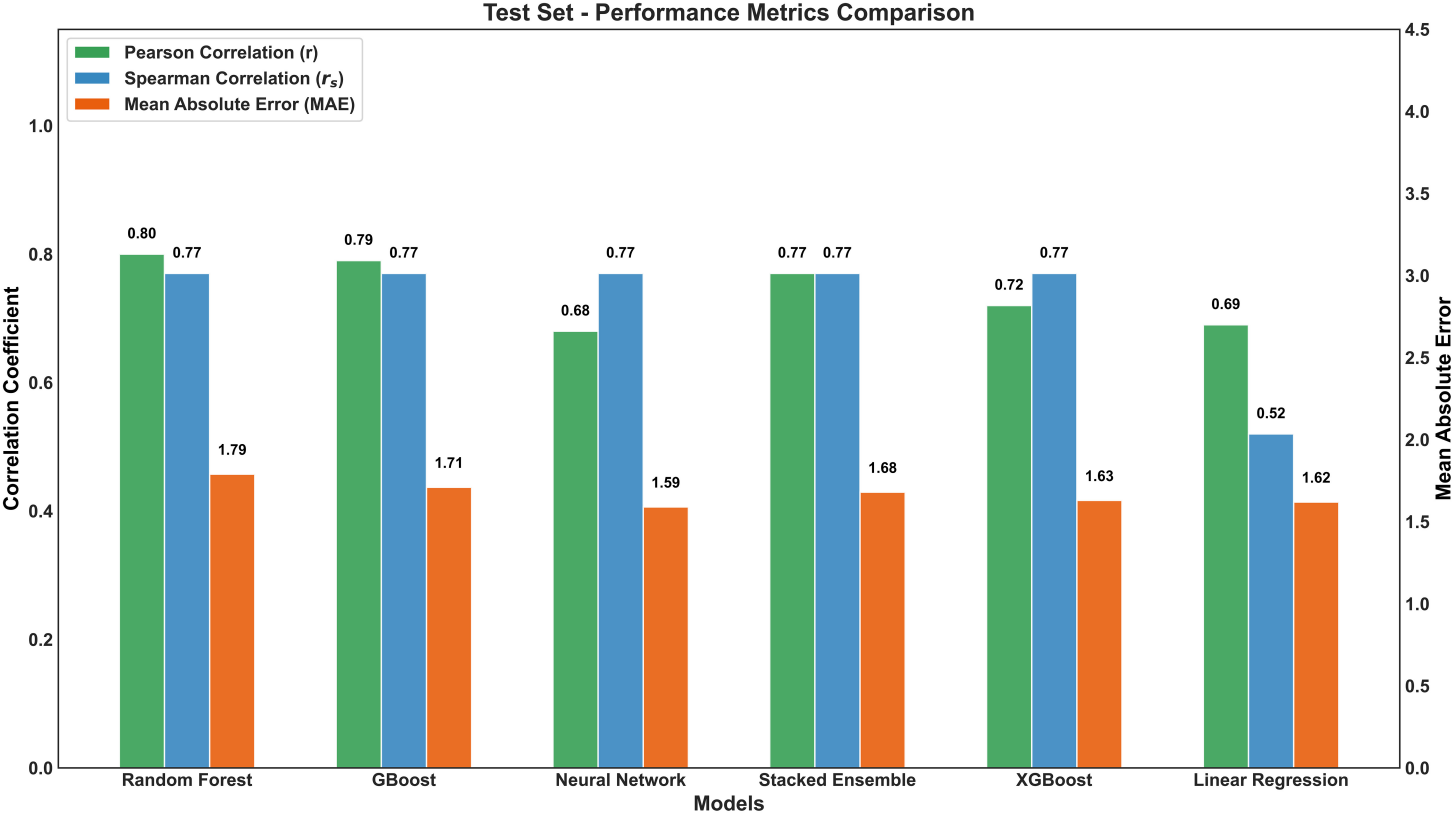
Performance comparison of machine learning models based on Pearson correlation (*r*), Spearman correlation (*r*_*s*_), and mean absolute error (MAE) for the test set. Random Forest Regression demonstrates the highest predictive accuracy, while Linear Regression shows the lowest performance.

Figure 6 and Table 1, we summarize the obtained results (linear correlation between model-specific PANTHER Scores and experimental Δ*G*, data shown in Supplemental Table S3) and further highlight the similarity in the performances reached by the tested ML approaches through the test set. In more detail, with these additional parameters we confirm the following few points: (1) the nearly identical and rather satisfactory performances of both RFR and GBoost methods with (*r*) of 0.80 and 0.79, with ρ-value of 0.04, respectively; (2) the notably encouraging performances by neural networks showing the lowest MAE value of 1.59 kcal/mol with the lowest (*r*) of 0.68; and (3) the relatively poor performances of linear regression as evidenced by its (*r*) of 0.69, *r*_*s*_ of 0.52, and ρ-value of 0.22. Overall, the analysis of mean absolute errors indicates low and comparable values across models, effectively ruling out markedly inaccurate predictions. It should be noted, however, that MAE values have limited relevance since our proposed methodology was developed to reach a convenient relationship between PANTHER Score and experimental Δ*G* values, which does not necessarily imply a numerical agreement between them. In other words, this study is not focused on predicting the absolute Δ*G* values; rather, we introduce a new fragmental methodology (local-to-global integration) to calculate a score (in kcal/mol), which satisfactorily aligns with the experimental values. Interestingly, the Spearman correlation for all the models except for Linear Regression (lowest *r*_*s*_ = 0.52) has high correlation of ranks with the value 0.77. This shows that almost all the models are consistent in reproducing promising local energy values.

Taken together, the model-specific PANTHER Scores and statistical analyses (ρ-value < 0.04) demonstrate that the developed ML model performances are reliable; however, the optimal ML model for final implementation into the PANTHER Scoring system remains to be identified. The next section focuses on the ML model selection process by considering the performance on the stress set.

### ML model assessment

To better evaluate the obtained models and considering the very similar performances reached when analyzing the test set, we collected an extended set of 110 protein–RNA complexes that meet the predefined criteria (detailed in Materials and Methods subsection “Data sets selection and preparation”). They were utilized to calculate the corresponding PANTHER Scores, which were compared with the respective experimental Δ*G* values. Notably, unlike the training and test data sets, we refrained from applying structural curation or performing MD simulations on the complexes of this additional data set that we refer to as the stress set (see Supplemental Table S4). This stress set was specifically designed to challenge our models and facilitate an unbiased evaluation under realistic conditions, ensuring that our assessment reflects true predictive performances in practical applications. This challenging stress test reveals clear performance differences between models, allowing the best one to be selected. Additionally, testing with untreated structure evaluates the dependence of the computed Score on structure preparation and provides a clear assessment of the score's transferability to novel protein–RNA systems.

**FIGURE 7.**
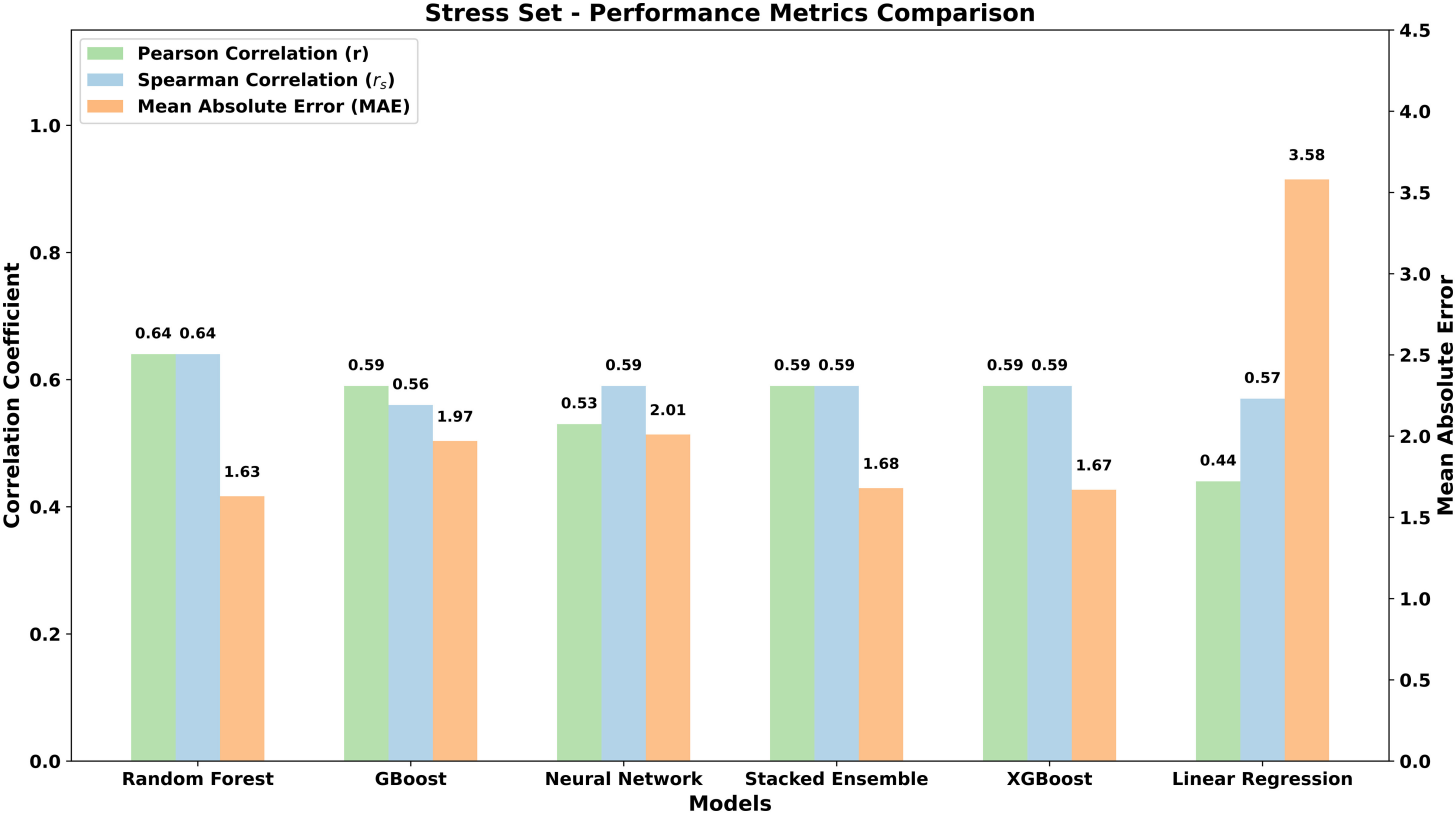
Performance comparison of machine learning models based on Pearson correlation (*r*), Spearman correlation (*r*_*s*_), and mean absolute error (MAE) for the stress set. Random Forest Regression demonstrates the best predictive accuracy with the highest (*r*) of 0.64 and the lowest MAE of 1.63. Linear Regression shows poor correlations and high MAE with respect to other models.

During stress assessment, we applied the same evaluation framework as done for the test set (see above) to an independent stress set with 110 complexes, which contained a larger and more diverse collection of molecular complexes. Figure 7 and Table 2 summarize the results obtained for the stress set and confirm, on average, satisfactory results with differences between the performances of the models in agreement with those monitored for the test set. In detail, the RFR model again confirms reaching the best performances in terms of both (*r*) = 0.64 with the lowest MAE of 1.63 and *r*_*s*_ (0.64) as validated by the lowest ρ-value (6.02 × 10^−14^), which suggests that its predictive capabilities are unlikely to have occurred by chance.

**TABLE 2.** Performance metrics of various machine learning models on the stress set when correlating the PANTHER Scores with known experimental Δ*G* values

Model	Stress set			
	*r*	MAE	*r*_*s*_	ρ-value
Random Forest Regression	0.64	1.63	0.64	6.02 × 10^−14^
GBoosting Regression	0.59	1.97	0.56	1.34 × 10^−10^
Neural Network	0.53	2.01	0.59	7.03 × 10^−12^
Stacked Ensemble	0.59	1.68	0.59	3.02 × 10^−12^
XGBoosting Regression	0.59	1.67	0.59	8.54 × 10^−12^
Linear Regression	0.44	3.58	0.57	3.02 × 10^−12^
PredPRBA	0.18	2.16	0.19	0.05
PRA-Pred	0.18	2.10	0.25	9.19 × 10^−3^

Metrics include Pearson correlation coefficient (*r*), mean absolute error (MAE), Spearman's rank correlation coefficient (*r*_*s*_), and ρ-value. Random Forest Regression demonstrates the highest predictive accuracy on both data sets. The table also includes the performance achieved by the other two tested methods (i.e., PredPRBA and PRA-Pred), which will be discussed in the subsection “Comparison of PANTHER Score with existing functional software.”

**FIGURE 8.**
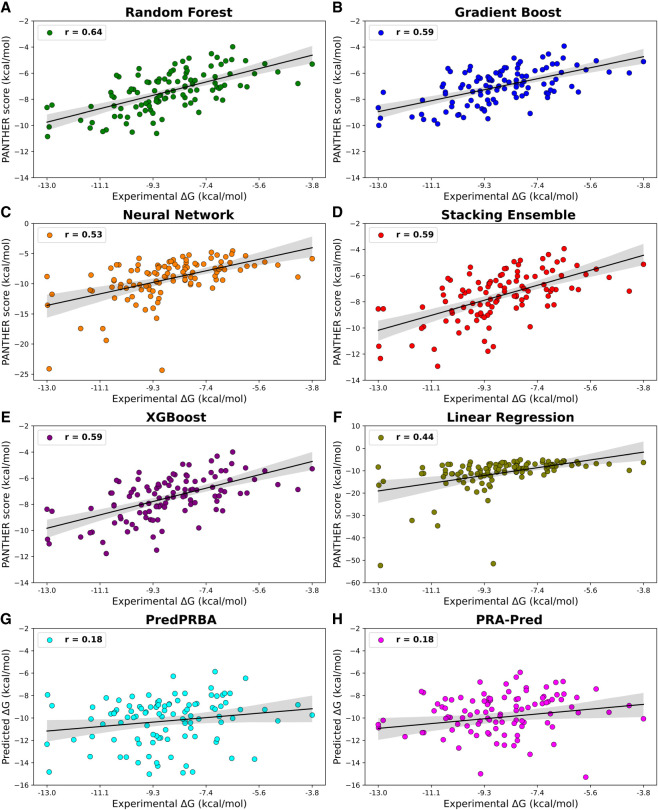
Scatter plots showing the linear correlations (as expressed by [*r*]) between experimental Δ*G* values and computed PANTHER Scores for various models: Random Forest Regression, Gradient Boost, Neural Network, Stacking Ensemble, XGBoost Regression, and Linear Regression. This figure also includes the corresponding plots for the other two tested methods (PredPRBA and PRA-Pred).

Figure 8 shows the plots of the model-specific PANTHER Score (kcal/mol) versus the experimental Δ*G* values (kcal/mol) and highlights the satisfactory performances reached by RFR, followed by the very similar results yielded by Gradient Boost, XGBoost, and Stacking Ensemble, while Neural Network and Linear Regression confirm their lower performances with some complexes, which behave as clear outliers. Similar performances provided by RFR, Gradient Boost, XGBoost, and Stacking Ensemble are also explained by considering that the computed scores are highly intercorrelated as can be seen from Supplemental Table S4, while these interrelations decrease when considering the scores from Neural Network and Linear Regression. All the linear equations for the correlations reported in Figure 8 show slopes far from being equal to 45° and intercepts ≠ 0 (data not shown) but, as discussed above, the relevance of the PANTHER Score is its encouraging correlation with experimental Δ*G* values regardless of the numerical agreement between these values.

**FIGURE 9.**
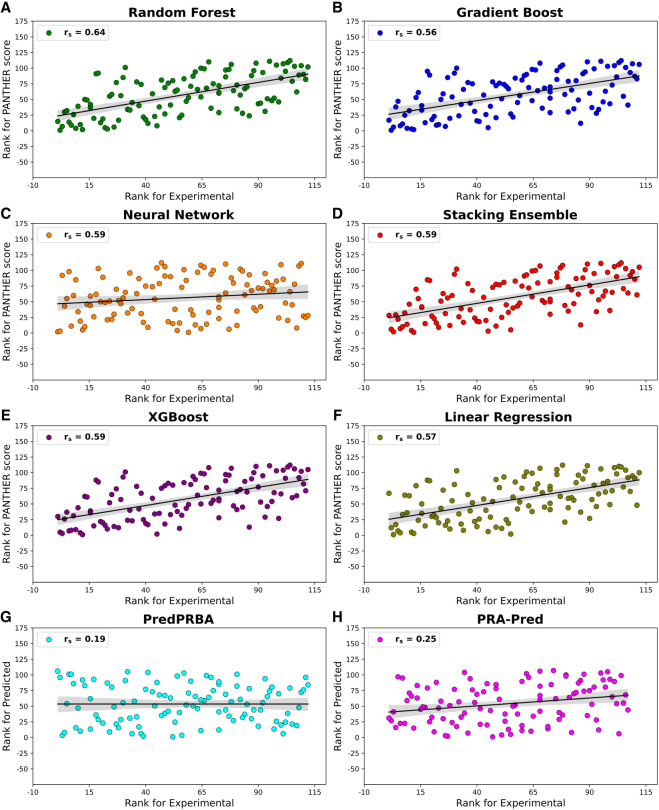
Scatter plots showing the Spearman rank correlation (*r*_*s*_) between experimental Δ*G* ranks and ranks of PANTHER Score for the models. This figure also includes the corresponding plots for the other two tested methods (PredPRBA and PRA-Pred).

The performance of the various ML models discussed in Figure 8 is further supported by the analysis of rank correlations between model-specific PANTHER Scores and experimental Δ*G* values, evaluated using Spearman's correlation. As evidenced by the corresponding Spearman rank correlation values, the RFR model is confirmed to be the best performing one with *r*_*s*_ (0.64), followed by Gradient Boost, XGBoost, Neural Network, and Stacking Ensemble, which reveal *r*_*s*_ value (0.59) as can be observed in Figure 9. Notably, when analyzing the rank correlation, the linear correlation also provides similar performances (*r*_*s*_ = 0.57). These results indicate positive strength and direction of association between experimental Δ*G* and score values, a very important outcome since PANTHER Score will be primarily used to rank and prioritize the analyzed RNA–protein complexes. Finally, all machine learning models demonstrated highly significant correlations with experimental Δ*G* values exhibiting ρ-values <10^−10^ to emphasize that these performances cannot occur by chance.

While the correlation and significance values of several models specifically RFR, GBoost, Stacked Ensemble, and XGBoost appear to be similar, this convergence reflects the robustness of the data set and the comparable learning capacity of tree-based algorithms applied to the same descriptors. Nevertheless, Figures 8 and 9 and Table 2 reveal that RFR displayed the best overall performance, with the highest correlation coefficients (*r* = 0.64, *r*_*s*_ = 0.64) and the lowest MAE (1.63 kcal/mol), outperforming the next-best GBoost model (*r* = 0.59, *r*_*s*_ = 0.56, MAE = 1.97 kcal/mol) by ∼8%–10% in correlation and 17% in prediction accuracy. Furthermore, its highly significant ρ-value (6.02 × 10^−14^) supports the reliability of this result. Overall, these findings indicate that, although several ensemble models perform comparably, RFR emerges as the most accurate and stable predictor and was therefore selected for the final implementation in the PANTHER Scoring system. Hereafter, the term “PANTHER Score” refers to the value obtained by combining the local interaction energies between amino acid–nucleotide base pairs, extracted from the here developed RFR ML model, and later processed by the local-to-global integration approach. In this workflow, the local interaction energies are first predicted by the ML model and then integrated using the local-to-global method to compute the final PANTHER Score. This whole process is discussed again with an example in the following subsection “Demonstration of the Random Forest Regression predictions for PANTHER Score calculation.”

#### Permutation feature importance analysis

To assess whether model predictions are driven by meaningful features, we performed permutation importance analysis on the test set for the two numerical features: distance and number of hydrogen bonds (Supplemental Figs. S4, S5; Supplemental Tables S7, S8). For each feature, we conducted 30 independent permutations where feature values were randomly shuffled across all pairwise interactions, and the pairwise local energies were predicted using the RFR model and converted to PANTHER Score using our local-to-global method. Performance degradation was assessed relative to baseline using RMSE and Pearson correlation coefficient (*r*). Distance showed strong importance: permuting it increased RMSE by 0.140 ± 0.014 kcal/mol (+7.6%) and reduced Pearson (*r*) by 0.135 ± 0.013 (reported relative to [*r*] ≈ 0.80, ∼16.9% loss; both ρ < 10^−6^, one-sided *t*-test, mean ± SD over 30 permutations). The number of hydrogen bonds had an even larger impact on absolute error, increasing RMSE by 0.259 ± 0.007 kcal/mol (+14.0%), and reduced Pearson correlation coefficient (*r*) by 0.083 ± 0.011 (∼10.4% loss; *P* < 10^−6^). These results align with molecular recognition: Distance captures the distance-dependent nature of intermolecular forces and contributes most to rank-order discrimination across complexes (larger Δ*r*), while number of hydrogen bonds reflects hydrogen-bonding networks and contributes to the magnitude of predicted PANTHER Score (larger ΔRMSE). Together, they capture complementary aspects of protein–RNA binding thermodynamics.

#### Comparison of PANTHER Score with existing functional software

The performances achieved by all the model-specific PANTHER Scores were compared with those yielded by two similarly tailored available methods known as PredPRBA and PRA-Pred. These two methods were chosen since they are recent tools that are easily usable through accessible web tools. Notably, both methods accept the PDB ID as input, and thus the 110 complexes of the stress set were directly submitted without requiring any preparation step.

The results obtained for these two methods, which are reported in Table 2, Supplemental Table S4, Figures 7 and 8, reveal very poor performance if compared to those achieved by the PANTHER Score. In detail, the performances of PredPRBA and PRA-Pred are characterized by very low (*r*) (both 0.18) and *r*_*s*_ (0.19 and 0.25, respectively) values, which seem to indicate a lack of reliable correlations between the experimental Δ*G* values and the scores predicted by both methods, when analyzing the stress set of 110 untreated complexes from various categories. Also, the mean absolute errors (MAEs) exhibit rather high values (2.16 and 2.10 kcal/mol for PredPRBA and PRA-Pred, respectively), and the ρ-values (>10^−3^) suggest a limited significance of these correlations. Collectively, these comparative analyses further underline the notable performance achieved by the PANTHER Score since the proposed fragmental approach outperforms the other available tested tools.

To understand the substantial performance gap between PANTHER Score and existing methods, we analyzed the key differences in their underlying methodologies and training strategies.

PredPRBA trained Gradient Boosted Regression Tree (GBRT) models on 103 nonredundant protein–RNA complexes using 37 features. Although these features are structure-based, they represent global or interface-averaged properties, such as secondary structure content, solvent-accessible surface area, RNA base pair frequencies, and predicted folding energies, rather than explicit residue-to-nucleotide interactions or pairwise energetics. While the GBRT framework effectively captures statistical correlations between these global descriptors and binding affinity, it is unable to distinguish atom-level energetic contributions as well as to account for conformational dynamics. This limitation constrains generalization to structurally diverse or untreated complexes.

On the other hand, PRA-Pred advanced structure-based modeling by assembling 217 nonredundant complexes and defining 17 structural descriptors. However, these descriptors are aggregated across entire binding interfaces rather than being resolved for individual residue–nucleotide pairs. Furthermore, the multiple linear regression (MLR) framework employs only four to eight interface-averaged variables per model and assumes simple linear additivity and therefore cannot represent the critical nonlinear, distance-dependent effects in RNA–protein binding. Although both methods achieved high performance on their respective benchmarking data sets, their accuracy declined substantially when applied to the independent stress set.

In contrast, PANTHER Score introduces a two-tier, physics-aware framework that fundamentally differs from both approaches. At the local level, MD simulations address data scarcity and quality limitations by generating time-averaged pairwise interaction energies. Specifically, only interactions persisting for more than 70% of simulation time are retained, thereby filtering transient and nonspecific contacts. Each persistent residue–nucleotide pair is then decomposed into van der Waals and electrostatic components, providing physically meaningful, atom-level training data. Additionally, PANTHER Score employs RFR to predict local energies from structural features. In contrast to the linear MLR framework used in PRA-Pred, RFR can capture complex nonlinear relationships and feature interactions inherent in protein–RNA binding, thus leading to improved predictive accuracy for local interaction energies. Then, predicted local energies are integrated to estimate overall binding affinity with our local-to-global approach. This design offers three key advantages. First, it captures explicit residue–nucleotide energetics rather than interface-averaged properties, thereby preserving spatial resolution. Second, it leverages MD-derived dynamic data, enabling the model to learn physically derived relationships between pairwise features and interaction energies that would be inaccessible from static structures alone. Third, the two-stage framework of local energy prediction followed by local-to-global integration facilitates generalization across diverse RNA and protein types, as local interactions represent transferable building blocks for binding affinity predictions.

#### Demonstration of the Random Forest Regression predictions for PANTHER Score calculation

**FIGURE 10.**
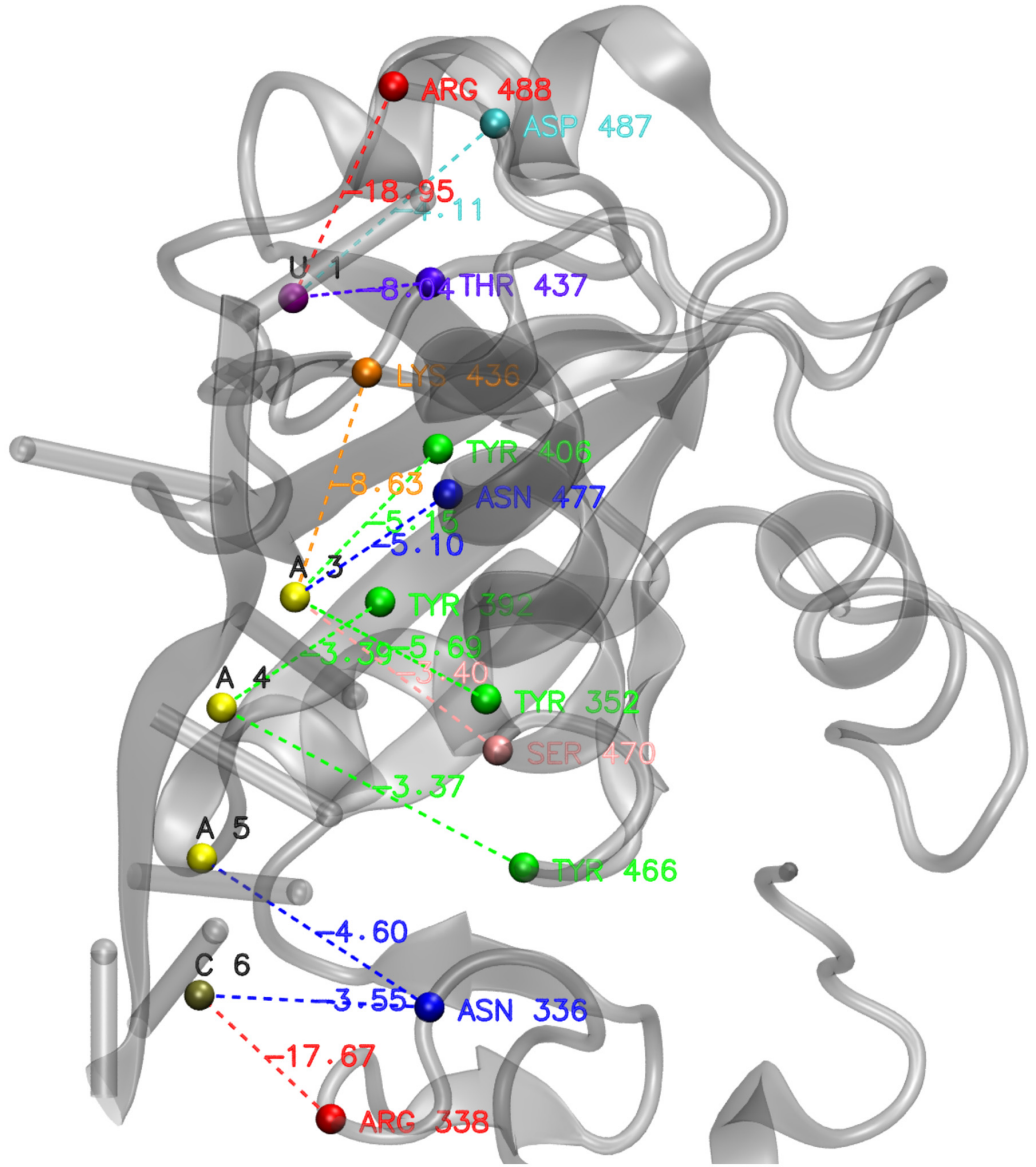
Visualization of protein–nucleotide interactions for the Mmi1 protein in complex with 7-mer RNA (PDB ID 6FPQ) showing residue pairs with their local binding energies (kcal/mol). Residue labels are positioned close to their corresponding spheres, with energy values displayed along dashed connection lines. These spheres are the center of mass of each interacting residue. By averaging these local energies obtained from Random Forest Regression models, we finally achieved the PANTHER Score of −7.05 kcal/mol, whereas the experimental value of 6FPQ is −6.93 kcal/mol. The interacting groups are colored according to their stabilizing energies by a color ramp ranging from red (strongly interacting groups) to blue (poorly interacting groups). The data are shown in Supplemental Table S6.

To illustrate the local-to-global approach of the PANTHER Score, we analyzed the structure of *S. pombe* Mmi1 in complex with 7-mer RNA complex (PDB ID: 6FPQ, experimental Δ*G* = −6.93 kcal/mol). Figure 10 shows the 3D structure of the complex, highlighting the key local interactions between amino acid and nucleotide bases (e.g., ASN-336 with A-5, ARG-338 with C-6). Each local interaction energy, as predicted by our RFR model, is listed in Supplemental Table S6, while an input example for RFR model has been detailed in Supplemental Table S5. Notably, the RFR model accurately estimates these local energies without requiring computationally expensive MD simulations, demonstrating the efficiency of the here developed approach. For instance, ARG-488 and U-1 exhibit a strong local energy of −18.95 kcal/mol, while other pairs like TYR-352 and A-3 contribute −5.69 kcal/mol. By averaging these local energies, we obtained a PANTHER Score of −7.05 kcal/mol, closely matching the experimental Δ*G* value (difference: −0.12 kcal/mol). This close agreement is a clear example of the predictive power of our approach, where ML-predicted local energy contributions effectively replace MD-derived local energies, maintaining satisfactory predictive accuracy. The graphical representation of Figure 10 and the predicted energy values (Supplemental Table S6) underscore the explainability of the PANTHER Score, which allows evaluating protein–RNA binding recognition processes through a computationally efficient framework.

### Overall assessment

In this study, we introduced the PANTHER Scoring function, a machine learning–based model developed to calculate scores specially tailored for protein–RNA interactions with high accuracy and reliability. By leveraging pairwise interaction features derived from MD simulations and a diverse data set of protein–RNA complexes, PANTHER Score demonstrated superior performance compared to existing methods, including PredPRBA (Deng et al. 2019) and PRA-Pred (Harini et al. 2024), as evidenced by its robust correlation coefficients and reduced prediction errors.

Among the models tested, those generated by RFR emerged as the most reliable ones, achieving the highest model-specific PANTHER Score versus experimental Δ*G* Pearson correlation coefficient (*r* = 0.80) and a mean absolute error (MAE = 1.79 kcal/mol) on the test set. Even under the challenging conditions when exposed to the stress set, RFR maintained its robustness, with (*r*) = 0.64 and MAE = 1.63 kcal/mol, highlighting its ability to handle unseen and untreated data. These findings underscore the strength of the here developed RFR model in capturing complex, nonlinear relationships inherent in protein–RNA interactions.

Thus, our approach demonstrates the value of employing a local-to-global methodology to generate high-quality data for model training. By calculating local energies purposely extracted from MD simulations and filtered by implementing noise reduction techniques (as detailed in Materials and Methods subsection “Extraction of pairwise local energies and noise reduction technique”), we produced a large and reliable data set of intermolecular interactions to effectively train the models. Such an approach shows several advantages, which can be summarized as follows. First, to reduce the high-frequency oscillations typically observed in MD simulations, interaction energies are averaged over 10 consecutive frames, and only those interactions that persist during 70% of the simulation period were considered. This lifetime cutoff and averaging process reduces random or negligible interactions and enhances the reliability of the local data set by focusing on the most stable interaction energy profiles. Second, to ensure comprehensive temporal coverage of the simulation data, a frame-skip interval of 40 frames is implemented between the 10-frame analyzed windows. This strategy effectively reduces the risk of oversampling from highly correlated adjacent frames, which could lead to biased results. By maintaining a balanced sampling approach, the interaction data reflect a broader spectrum of the system's dynamics throughout the entire simulation time. Third, the combined methodology of averaging interaction energies and implementing frame-skipping effectively captures the harmonic fluctuations inherent in the system. This dual approach strikes a balance between preserving detailed local interaction data and encompassing broader temporal dynamics. Consequently, it enhances the data set's capacity to reveal significant trends and patterns in molecular interactions over time. Fourth, the resulting data set, characterized by average and temporally spaced interaction energies, is particularly suited for training machine learning models. The balanced structure of the data set improves the generalizability of the trained models by exposing them to a diverse array of interaction patterns across various time frames. Finally, the 12 Å cutoff distance is chosen to encompass biologically relevant short-range interactions, such as ion-pairs, hydrogen bonding, and van der Waals forces, while effectively excluding long-range interactions that negligibly contribute to pairwise energy calculations. This criterion ensures that the analysis remains focused on interactions that are significant from a biophysical perspective. Altogether, this approach allowed us to overcome limitations posed by the scarcity of experimental Δ*G* values and enhance the predictive reliability of PANTHER Score.

Remarkably, such a fragmental approach can have a general role and can find fruitful application to analyze the intermolecular interactions in protein–protein complexes or even in ligand–protein complexes. Moving forward, the approach is flexible and expandable by incorporating additional protein–RNA complexes to enhance the score's predictive power by exploring more interaction features. These efforts will further refine PANTHER Score's predictive capabilities, broadening its applicability to other nucleic acid–protein systems and advancing our understanding of these essential biological interactions.

## MATERIALS AND METHODS

### PANTHER Score

In this study, we developed PANTHER Score, a scoring system designed to estimate protein–RNA binding affinities through an integrated, multistep computational strategy. The workflow involves several sequential steps. First, a curated data set of RNA–protein complexes was selected to ensure the inclusion of systems with known experimental binding free energies (Δ*G*), strategically increasing the number of complexes to tackle the lack of experimental data for developing an effective ML model. Next, MD simulations were performed on representative complexes to generate physically realistic conformations and extract energetic and structural data (further detailed in Materials and Methods subsection “Extraction of pairwise local energies and noise reduction technique”). From these trajectories, pairwise decomposed interaction energies between amino acid residues and nucleotide bases were calculated, providing a detailed description of the local energetic landscape at the binding interface. These pairwise energies and structural descriptors were then used to train multiple regression machine learning (ML) models to estimate the binding affinity of protein–RNA complexes by combining machine-learning-derived local energy predictions with our proposed local-to-global integration method. The workflow thus operates in two main stages:

- Local energy prediction: Multiple regression ML models, including RFR, Gradient Boosting Regression, Extreme Gradient Boosting, Linear Regression, Stacked Ensemble, and Neural Network, were trained using the MD-derived amino acid–nucleotide pairwise local energy data, enabling the prediction of local interaction energies for any given amino acid–nucleotide pair from distance-weighted structural descriptors.
- Local-to-global integration: The predicted local energies are then aggregated through a distance-weighted summation, where each interaction contributes according to its spatial proximity and relevance. This integration produces the PANTHER Score (kcal/mol), an overall binding affinity of protein–RNA complexes.

This local-to-global framework effectively combines atomistic simulation data with ML-derived predictions to produce an interpretable and generalizable measure of protein–RNA binding energetics. Notably, to define the final PANTHER Score, we rigorously evaluated all the ML models through test set and stress set, and we finally selected only one model (refer to Results and Discussion subsection: “ML model assessment” for further details) to produce an interpretable and generalizable measure of protein–RNA binding energetics.

The following sections describe each step of the PANTHER Score workflow in detail.

### Data set selection and preparation

In this study, a total of 163 RNA–protein complexes were retrieved from the Protein Data Bank (PDB) (Berman et al. 2000), analyzed (refer to Supplemental Tables S1–S4), and subsequently subdivided into three distinct data sets for the training, testing, and stress testing phases of the ML model. These 163 protein–RNA complexes were selected from a combined pool of 1116 protein–nucleic acid complexes, integrating data from PRBAB v2.0 (Hong et al. 2023), PDBbind (Wang et al. 2004), ProNAB (Harini et al. 2022), and the data set from Nithin et al. (2019). The selection criteria for these complexes included the following: (1) three-dimensional structures resolved through X-ray crystallography or NMR with a resolution better than 3.0 Å, (2) the inclusion of only single-stranded RNA, (3) the exclusion of inhibitors and metal ions, (4) the presence of standard residues and nucleotide bases exclusively, (5) the omission of zinc-finger proteins, (6) an even distribution of the four nucleotide bases in RNA, and (7) a diverse representation across various protein classifications and organisms. The selected complexes were then filtered by removing redundancy using Clustal Omega (Sievers et al. 2011), with a 70% sequence identity threshold to collect a total of 163 protein–RNA complexes.

By comparing our data set with existing resources, including PRBAB v2.0 (Hong et al. 2023), PDBbind (Wang et al. 2004), ProNAB (Harini et al. 2022), and the data set from Nithin et al. (2019), we found experimental Δ*G* values for 130 (out of 163) complexes. To effectively divide the data set for ML applications, we considered that the initial training of ML models is based only on MD simulations, while the following validation of the PANTHER Score requires complexes with experimental Δ*G* values. Thus, the training set comprised all 33 complexes without experimental Δ*G* values (training set 2) plus 13 complexes with experimental Δ*G* values (training set 1) selected to satisfactorily cover the protein–RNA interaction space, as schematized in Figure 1. Among the remaining 117 complexes with experimental Δ*G* values, we randomly selected seven complexes that constitute the test set for preliminary tuning and validation of both ML models and ML-derived PANTHER Scores. All the 53 complexes belonging to training (set 1 + set 2) and test sets underwent MD simulations. Notably, the 20 complexes (13 from training set 1 + 7 from test set) with known experimental Δ*G* values, which underwent MD runs were also exploited to initially assess the reliability of the here-adopted local-to-global approach.

Finally, the remaining 110 complexes with known experimental Δ*G* values were assigned to the stress set, which was used to assess the predictive capability of each ML model-specific PANTHER Score and to identify the most accurate model for final implementation. Notice that, unlike the train and test sets, the complexes of the stress set did not undergo MD runs and postprocessing of the extracted data but were used to directly extract the interaction features required by the ML models.

Importantly, the here-adopted local-to-global methodology and the so-organized data sets offer some relevant advantages. They allow the following: (1) combining complexes with and without experimental Δ*G* values for the training phase, while complexes with experimental Δ*G* values were used only for the test and validation phases; (2) minimizing the computational cost by carrying out MD runs only on 53 complexes and not on all 163 collected systems; and (3) augmenting the data to be used for training ML models by considering pairwise local interaction energies between amino acids and nucleotide bases as derived from MD simulations.

Prior to MD simulations, all complexes underwent a careful structural curation process to model missing amino acids or atoms using VEGA ZZ (Pedretti et al. 2004), Modeler (Fiser et al. 2000), and Chimera (Pettersen et al. 2004) programs. The 53 curated structures of training and test sets underwent MD simulations to derive pairwise interaction energies (further described in subsection “Extraction of pairwise local energies and noise reduction technique”).

For training the ML models, a total of 87,117 MD-derived pairwise interactions, each associated with its corresponding local energy, were used as the training data set. Once the models were trained, 18,971 additional MD-derived pairwise interactions were used to evaluate how well the ML-predicted local energies reproduced the original MD-derived values. These ML-derived local energies were then integrated using the local-to-global methodology to compute the model-specific PANTHER Scores.

For the stress set, the procedure differed slightly. Instead of relying on expensive MD-derived time-averaged interaction energy approach data (described in subsection “Extraction of pairwise local energies and noise reduction technique”), we used only the raw structural information from the PDB files as input. The input features consisted of the pairwise interaction descriptors, as illustrated in Supplemental Table S5. The trained ML models were then used to predict the local interaction energies directly from these structural inputs, and the resulting local energies were subsequently integrated through the local-to-global approach to generate the PANTHER Scores for each model.

### Molecular dynamics simulation

The curated 53 complexes underwent MD simulations using the Amber20 suite (Case et al. 2023) with the AMBER14SB (Tian et al. 2020) (for proteins) and OL3 force-fields (Zgarbová et al. 2011) (for RNA), the TIP3P water model (Jorgensen et al. 1983), and embedding the system into a cubic box. The LEaP algorithm (Case et al. 2023) was used to generate coordinate and topology files for AMBER. To neutralize the systems, Na^+^ or Cl^−^ counterions were added as needed, ensuring that all ions remained in the solvent and at least 10 Å away from the protein–RNA binding region. Hence, all the complexes underwent an initial minimization during which the hydrogen atoms were first minimized, then the whole complex was minimized, and finally the water molecules were minimized. Next, each system was gradually heated from 0 to 300 K using a velocity-rescaling algorithm. Lastly, the equilibration phase was organized as follows: (1) each system underwent a 200 psec isobaric–isothermal ensemble (NPT ensemble) (Andersen 1980) with 1 bar of pressure, (2) followed by five consequent 100 psec MD runs with the NPT ensemble to gradually adjust the volume of the system to attain water density within the water cubic box equal to 1 g/cm^3^, and (3) a short 100 psec MD run with the isothermal–isochoric ensemble (NVT ensemble) (Alder and Wainwright 1959) was carried out to relax all the atoms before production. During the process, all bond lengths were kept fixed using the LINCS algorithm (Hess et al. 1997), thus allowing an integration step of 2 fsec. The particle mesh Ewald method (Darden et al. 1993) was used to compute long-range interactions above a cutoff radius of 1.1 nm. The last step of MD simulations involved the production run of 500 nsec with the NVT ensemble (Alder and Wainwright 1959) to generate the corresponding trajectories, which include a saved frame every 50 psec for a total of 10,000 frames per trajectory.

### Extraction of pairwise local energies and noise reduction technique

Pairwise decomposition involves calculating the energy contributions between specific atoms or groups of atoms. Here, the interaction energy between an amino acid (*i*) and a nucleotide base (*j*) is equal to the sum of the van der Waals (*E*_vdw_) and electrostatic (*E*_elec_) contributions between all atom pairs of the two interacting groups:

$$E_{ij}=E_{\mathrm{vdw},ij}+E_{\mathrm{elec},ij}$$

The van der Waals interactions (*E*_vdw,*ij*_) were calculated by the Lennard-Jones potential for all atom pairs (*a* ɛ *i*, *b* ɛ *j*).

$$E_{\mathrm{vdw},ij}=\underset{a\varepsilon i\;}{\sum }\underset{b\varepsilon j}{\sum }4_{\varepsilon ab}\left[{\left(\frac{\sigma_{ab}}{r_{ab}}\right)}^{12}-{\left(\frac{\sigma_{ab}}{r_{ab}}\right)}^{6}\right],$$

where *ɛ*_*ab*_ is the depth of the potential well for the interaction between atom *a* and atom *b*, σ_*ab*_ is the distance at which the potential between *a* and *b* is zero or the minimum, and *r*_*ab*_ is the distance between atom *a* (in group *i*) and atom *b* (in group *j*). The 12th term and the sixth term represent the repulsive and attractive terms, respectively.

The electrostatic energy component is calculated by Coulomb's law.

$$E_{\mathrm{elec},ij}=\underset{a\varepsilon i\;}{\sum }\underset{b\varepsilon j}{\sum }\frac{q_{a}q_{b}}{4\pi \varepsilon_{0}r_{ab}},$$

where *q*_*a*_ and *q*_*b*_ are the partial charges on atoms *a* (in group *i*) and *b* (in group *j*), respectively, *ɛ*_0_ is the relative dielectric constant in implicit solvent, and *r*_*ab*_ is the distance between atom *a* and atom *b*.

To reduce computational costs, a cutoff distance (*r*_cut_) of 12 Å was applied for pairwise decomposition energy calculations. These calculations were conducted to analyze the interactions between amino acids and nucleotide pairs only if the distance between their respective centers of mass (COMs) satisfied *r*_*ab*_ ≤ *r*_cut_. This threshold was chosen to be slightly larger than the 11 Å cutoff used with the particle mesh Ewald method (Darden et al. 1993). The additional 1 Å margin ensures that we can capture all significant interactions that might occur at the boundary of the PME cutoff. The selection of 12 Å is also justified by the exponential decay of electrostatic and van der Waals interactions beyond this threshold, which renders negligible the resulting energetic contributions. This approach encompasses both short-range and intermediate-range interactions, ensuring the inclusion of biologically relevant contacts while maintaining consistency with simulation parameters.

To consider the dynamic behavior of these interactions and to produce large-scale data for ML training, we adopted a time-averaged interaction energy approach by averaging the interaction energy between the amino acid *i* and the nucleotide base *j* over 10 frames:

$$<E_{ij}>=\frac{1}{N}\overset{N}{\underset{t=1}{\sum }}E_{ij}^{\left(t\right)},$$

where *N* is the averaging window and is equal to 10 and $E_{ij}^{\left(t\right)}$ is the interaction energy between residue *i* and nucleotide *j* at the time frame *t*. After calculating the average interaction energy for a set of 10 consecutive frames, we skipped the following 40 frames and repeated the calculations on the next 10-frame window. This process was iterated until the end of the 500 nsec simulation. By applying this method and for each interaction pair, we obtained a series of 10-frame averaged interaction energies.

The choice of a window of 10 frames spaced by 40 skipped frames should represent an optimal balance between statistical robustness and computational efficiency. Ten frames provide sufficient sampling to smooth thermal fluctuations while maintaining the ability to capture relevant interactions. The 40 skipped frames should avoid redundancies between computed averages, ensuring statistical independence between consecutive data points. This approach should filter out transient interactions by focusing on persistent binding modes and discarding those interactions that are present in only 20%–30% of the trajectory, as these represent random events rather than stable binding conformations. This filtering strategy should ensure that the ML models are trained on thermodynamically significant interactions, thus improving the biological relevance of their predictions.

Using the time-averaged interaction energy approach, we extracted a total of 331,744 amino acid–nucleotide pair interactions and their corresponding local energies from the MD trajectories of 46 protein–RNA complexes (training set 1 + training set 2). For the test set, an additional 58,000 pairwise interactions were obtained from the MD trajectories of seven protein–RNA complexes. To reduce noise and ensure data quality, we retained only those interactions that remained stable for at least 70% of the simulation time, thereby focusing on persistent and meaningful amino acid–nucleotide contacts. After this refinement, the data sets consisted of 87,117 and 18,971 interaction pairs for the training and test sets, respectively, each associated with its corresponding MD-derived local energy.

These refined, time-averaged interaction data were then used to train multiple ML models. Once trained, the models were applied to the test set to predict local interaction energies. The resulting 18,971 predicted local energy values were then grouped according to their corresponding PDB IDs, first allowing verification of the ML-derived data and later allowing the application of the local-to-global methodology to integrate the predicted local energies and compute the model-specific PANTHER Scores for each of the seven protein–RNA complexes of the test set. This workflow ensured the inclusion of diverse yet reliable interaction patterns while minimizing noise and avoiding oversampling from closely correlated trajectory frames.

Along with the above-mentioned energetic averages, a set of structural descriptors was collected to be used as features to train the ML models. They included (1) pairwise distances, quantified as the Euclidean distance between the centers of mass (Å) of the interacting amino acid and nucleotide and (2) various features for each pairwise interaction to better describe the relevant biophysical aspects of protein–RNA recognition and binding (as detailed in Supplemental Table S5).

### Development of prediction models

To predict protein–RNA local interaction energies, we explored a diverse range of regression models, starting with linear regression as a baseline and progressing to more complex approaches, including tree-based ensemble methods such as RFR (Ho 1995), boosting-based ensembles such as Gradient Boosting Regression (GBR) (Friedman 2001) and Extreme Gradient Boosting regression (XGBoost) (Chen and Guestrin 2016), as well as a Stacked Ensemble model (Wolpert 1992). The Stacked Ensemble combines the predictions of RFR, GBR, and XGBoost using a direct aggregation strategy. Additionally, we implemented a Neural Network model to further enhance predictive performance.

The selected models were chosen to balance interpretability, computational efficiency, and predictive accuracy. A standardized ML pipeline was developed in Python 3.9 using Scikit-Learn 0.24.1 (Pedregosa et al. 2011), Keras version 3.3.3, and TensorFlow version 2.16.1 libraries. Feature preprocessing was performed using Scikit-Learn's ColumnTransformer, ensuring consistency across all models. Numerical features (e.g., distance and number of hydrogen bonds) were standardized using StandardScaler, while categorical features (e.g., amino acid and nucleotide types) were one-hot encoded via OneHotEncoder. The data set was initially partitioned into training (80%) and validation (20%) subsets using train, test, split. To further evaluate model generalization, we applied a 10-fold cross-validation strategy (Kohavi 1995) exclusively on the training set, where in each fold, 90% of the training data were used for model fitting and 10% for validation. Hyperparameter tuning was performed via grid search (Pedregosa et al. 2011) over an extensive parameter space to optimize each model's configuration. Model performance was evaluated using Pearson's correlation coefficient (*r*), mean absolute error (MSE), Spearman's rank correlation coefficient (*r*_*s*_), and the statistical significance (ρ-value) (Zar 1972). Furthermore, learning and loss curves were monitored to ensure stable training and convergence. The Neural Network architecture was implemented using Keras version 3.3.3 and TensorFlow version 2.16.1, incorporating ReLU activation functions, L2 regularization, and the Adam optimizer to enhance model robustness and generalization.

### Local-to-global integration

Our approach to predict protein–RNA binding affinities exploits ML models and a local-to-global methodology, moving from local interaction energies to binding affinity predictions, which we call the PANTHER Score. This process consists of several steps that utilize molecular features to compute a comprehensive energy landscape. The workflow begins with identifying interacting amino acid–nucleotide pairs within a given protein–RNA complex using an in-house interaction pattern script. We analyze the structural data to detect significant interactions based on distance thresholds and binding patterns. Specifically, interacting amino acid–nucleotide pairs are identified by considering pairs of amino acid residues and nucleotide bases characterized by a distance between their centers of mass (COMs) within a cutoff threshold of 12 Å.

For each identified interaction pair, we extract features including amino acid type, nucleotide base identity, center-of-mass distance, and number of H-bonds (as shown in Supplemental Table S5). These features serve as input to ML models to predict the corresponding local pairwise interaction energies. The PANTHER Score (kcal/mol) is then determined through an integrative approach that aggregates these local interaction energies according to the following formula:

$${\mathrm{PANTHER}}_{\mathrm{score}}=\int_{\Omega }\rho (r)E(r)dr,$$

where *ρ*(***r***) represents the spatial density of interaction pairs at position *r*, *E*(***r***) is the local energy contribution at position *r*, and Ω is the binding interface (all areas where interactions occur). We sum up energy contributions from all interaction pairs, while considering their distribution in space. This integral can be discretized for computational implementation for energy prediction:

$${\mathrm{PANTHER}}_{\mathrm{score}}=\overset{n}{\underset{i=1}{\sum }}\omega_{i}E_{i},$$

where *ω*_i_ represents a weighting factor that adjusts each interaction's contribution, and *E*_*i*_ is the predicted energy for the *i*th amino acid–nucleotide pair. To ensure that closer interactions have greater contribution, *ω*_*i*_ is based on an exponential decay function:

$$\omega_{i}=\frac{\exp\left(-r_{i}/r_{0}\right)}{\sum_{\;j=1}^{N}\exp\left(-r_{i}/r_{0}\right)},$$

where *r*_*i*_ is the distance of the interaction pair, and *r*_0_ is a characteristic length scale of 9 Å. The negative sign ensures that the function decreases as distance increases. By applying this weighting function, interactions occurring at shorter distances (particularly below 9 Å) contribute more significantly to the total binding energy.

Finally, we refine our PANTHER Score by using a distance-dependent function:

$${\mathrm{PANTHER}}_{\mathrm{score}}=\frac{\sum_{i=1}^{n}\alpha (r_{i})E_{i}}{n},$$

where *α*(*r*) is a distance-dependent weighting function that typically decreases with increasing distance, reflecting the diminishing contribution of more distant interactions to the binding free energy. The resulting PANTHER Score, expressed in kcal/mol, offers an interpretable and accurate estimation of protein–RNA binding energetics, making it a valuable tool for understanding biomolecular recognition processes.

### Large-scale application

After completing the workflow to calculate the PANTHER Score, we aimed to automate its application to the protein–RNA complexes of interest. To achieve this, we developed two in-house scripts. The first script incorporates a parsing algorithm designed to read and extract data from PDB files. This also detects the hydrogen bonds by evaluating both the distance and angle to evaluate their stability. Specifically, the distance between the donor hydrogen atom and the acceptor atom must be <3.5 Å, while the angle should range from 120° to 180°. The position of the hydrogen atom was determined by the bond vector method, which assumes that the hydrogen atom lies along the bond vector extending from the donor atom toward the acceptor atom at a typical bond length (∼1 Å for N–H or O–H bonds).

The second script utilizes the input features derived from a raw PDB file (e.g., center of mass distance, amino acid and nucleotide base types, hydrogen bond count) as input for the ML model to predict pairwise local interaction energies without performing MD simulations. The predicted interaction terms are then aggregated to estimate the PANTHER Score for a given protein–RNA complex. This workflow was designed to enable efficient large-scale predictions and was applied to the stress set of 110 structures, showing interesting performances with a speed of ∼55 structures/min.

To evaluate the performance of our models, we conducted a comparative analysis (refer to Results and Discussion subsection “Comparison of PANTHER Score with existing functional software”) with the web-based tools PredPRBA (Deng et al. 2019) and PRA-Pred (Harini et al. 2024) on the stress set consisting of 110 protein–RNA complexes. In detail, we compared the correlation between the experimental Δ*G* and predicted binding affinity values for the three methods.

## DATA DEPOSITION

All the data produced are either shared in the main manuscript or in the Supplemental Material, which makes it easier for reproducibility and applicability with other methods.

## SUPPLEMENTAL MATERIAL

Supplemental material is available for this article.

## Figures and Tables

**Parisa Aletayeb:**
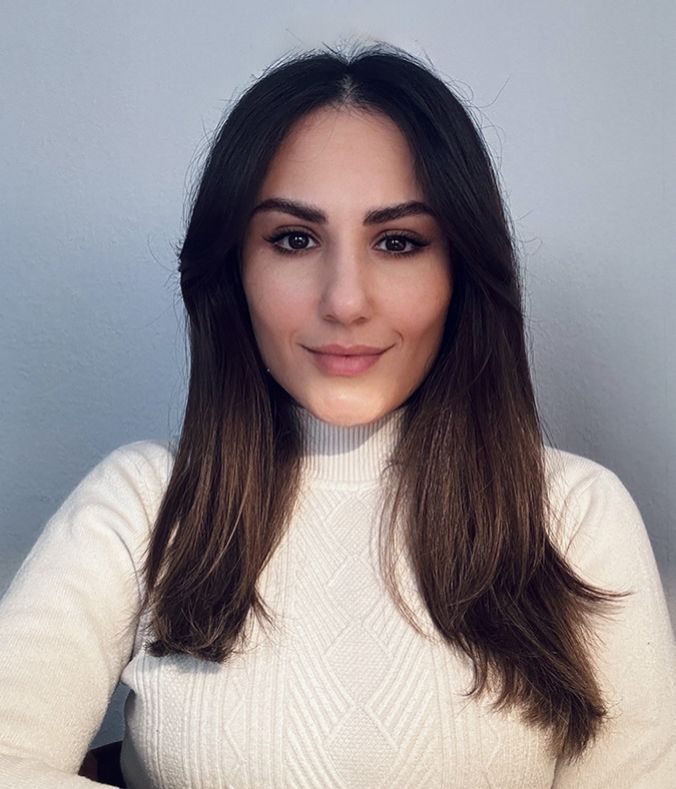


## MEET THE FIRST AUTHOR

***Meet the First Author(s)* is an editorial feature within *RNA*, in which the first author(s) of research-based papers in each issue have the opportunity to introduce themselves and their work to readers of *RNA* and the RNA research community. Parisa Aletayeb is the first author of this paper, “PANTHER Score: Protein-Affinity for Nucleic Target-binding, Hybridization, and Energy Regression.” Parisa is a PhD candidate in Pharmaceutical Sciences at the University of Milan. She completed her BSc in Molecular Genetics and MSc in Biochemistry before transitioning into molecular design and computational chemistry during her PhD, where she has been integrating her background in genetics and biochemistry with advanced computational modeling to develop new machine learning–based tools to address unmet challenges in RNA-based drug discovery.**

**What are the major results described in your paper, and how do they impact this branch of the field?**

In this study, we developed a novel scoring function, which directly addresses key limitations in protein–RNA binding affinity prediction: the lack of experimental energy of protein–RNA complexes, the low accuracy and reliability of existing models, and the use of static structures that ignore molecular dynamics. By integrating this new dynamic, fragment-based approach with machine learning, we created a model that delivers higher accuracy, stronger robustness, and better generalization than currently available tools in the market. Our results show that this combined strategy sets a new benchmark for predicting protein–RNA binding affinity in this area of research.

**What led you to study RNA or this aspect of RNA science?**

As the RNA therapeutics field expanded in recent years, we recognized a critical gap: the lack of reliable and accurate models for predicting RNA–ligand and RNA–protein binding affinities, a limitation that contributes to increased experimental demands and slows scientific progress. I viewed this not only as an unmet need but as a problem I could actively address. By moving beyond outdated, static methodologies and focusing on more innovative dynamic and data-driven approaches, I was motivated to develop new computational solutions that could better support and accelerate RNA-targeted drug discovery.

**During the course of these experiments, were there any surprising results or particular difficulties that altered your thinking and subsequent focus?**

One of the defining challenges of this project was the absence of any ready-to-use methodology. At every stage, from data generation to model construction, we had to design new procedures, evaluate their scientific validity, and test their practical feasibility. Our first major obstacle was ensuring that the model would be trained on the highest-quality data possible, which required capturing the “dynamic” nature of protein–RNA interactions. For this reason, we ran 500 nsec MD simulations for each complex, an approach that is both computationally intensive and resource-demanding. To make this feasible, we used GPU resources of the HPC facility.

Once the simulations were completed, we encountered the next unexpected challenge: the sheer volume and complexity of the decomposed pairwise energy data. The raw data set contained thousands of amino acid–nucleotide interactions, many of which were dominated by noise from transient or unstable contacts. This forced us to rethink our approach and develop a dedicated noise-reduction strategy, one that required extensive trial-and-error to balance statistical rigor with biological relevance. Although demanding, this process ultimately shaped the core of our methodology and significantly strengthened the final model.

**If you were able to give one piece of advice to your younger self, what would that be?**

I would remind myself that progress in science is rarely linear; uncertainty, failed attempts, and slow steps are part of the process. Stay persistent, ask for help when needed, and trust that clarity comes through iteration, not perfection.

**Are there specific individuals or groups who have influenced your philosophy or approach to science?**

I have been strongly influenced by my supervisor, Professor Alessandro Pedretti, and my mentor, Dr. Akash Deep Biswas, who emphasized both rigor and creativity in computational research. Their guidance taught me that high-quality science requires not only technical skill but also the willingness to explore unconventional solutions.

## References

[RNA080646ALEC1] Alder BJ, Wainwright TE. 1959. Studies in molecular dynamics. I. General method. J Chem Phys 31: 459–466. 10.1063/1.1730376

[RNA080646ALEC2] Andersen HC. 1980. Molecular dynamics simulations at constant pressure and/or temperature. J Chem Phys 72: 2384–2393. 10.1063/1.439486

[RNA080646ALEC3] Baek M, McHugh R, Anishchenko I, Jiang H, Baker D, DiMaio F. 2024. Accurate prediction of protein–nucleic acid complexes using RoseTTAFoldNA. Nat Methods 21: 117–121. 10.1038/s41592-023-02086-5 37996753 PMC10776382

[RNA080646ALEC4] Bell DR, Weber JK, Yin W, Huynh T, Duan W, Zhou R. 2020. In silico design and validation of high-affinity RNA aptamers targeting epithelial cellular adhesion molecule dimers. Proc Natl Acad Sci 117: 8486–8493. 10.1073/pnas.1913242117 32234785 PMC7165443

[RNA080646ALEC5] Berman HM, Westbrook J, Feng Z, Gilliland G, Bhat TN, Weissig H, Shindyalov IN, Bourne PE. 2000. The Protein Data Bank. http://www.rcsb.org/pdb/status.html. 10.1093/nar/28.1.235 PMC102472 10592235

[RNA080646ALEC6] Bheemireddy S, Sandhya S, Srinivasan N, Sowdhamini R. 2022. Computational tools to study RNA-protein complexes. Front Mol Biosci 9: 954926. 10.3389/fmolb.2022.954926 36275618 PMC9585174

[RNA080646ALEC7] Case DA, Aktulga HM, Belfon K, Cerutti DS, Cisneros GA, Cruzeiro VWD, Forouzesh N, Giese TJ, Götz AW, Gohlke H, 2023. AmberTools. J Chem Inf Model 63: 6183–6191. 10.1021/acs.jcim.3c01153 37805934 PMC10598796

[RNA080646ALEC8] Chen T, Guestrin C. 2016. XGBoost: a scalable tree boosting system. In KDD '16: Proceedings of the 22nd ACM SIGKDD International Conference on Knowledge Discovery and Data Mining, 13–17 August 2016, pp. 785–794, Association for Computing Machinery. 10.1145/2939672.293978

[RNA080646ALEC9] Darden T, York D, Pedersen L. 1993. Particle mesh Ewald: an N⋅log(N) method for Ewald sums in large systems. J Chem Phys 98: 10089–10092. 10.1063/1.464397

[RNA080646ALEC10] Deng L, Yang W, Liu H. 2019. PredPRBA: prediction of protein-RNA binding affinity using gradient boosted regression trees. Front Genet 10: 637. 10.3389/fgene.2019.00637 31428122 PMC6688581

[RNA080646ALEC11] Ding Z, Kihara D. 2018. Computational methods for predicting protein-protein interactions using various protein features. Curr Protoc Protein Sci 93: e62. 10.1002/cpps.62 29927082 PMC6097941

[RNA080646ALEC12] Feig AL. 2009. Studying RNA-RNA and RNA-protein interactions by isothermal titration calorimetry. Methods Enzymol 468: 409–422. 10.1016/S0076-6879(09)68019-8 20946780 PMC3035487

[RNA080646ALEC13] Fiser A, Do RKG, Šali A. 2000. Modeling of loops in protein structures. Protein Sci 9: 1753–1773. 10.1110/ps.9.9.1753 11045621 PMC2144714

[RNA080646ALEC14] Friedman JH. 2001. Greedy function approximation: a gradient boosting machine. Ann Stat 29: 1189–1232. 10.1214/aos/1013203451

[RNA080646ALEC15] Harini K, Srivastava A, Kulandaisamy A, Gromiha MM. 2022. ProNAB: database for binding affinities of protein–nucleic acid complexes and their mutants. Nucleic Acids Res 50: D1528–D1534. 10.1093/nar/gkab848 34606614 PMC8728258

[RNA080646ALEC16] Harini K, Kihara D, Michael Gromiha M. 2023. PDA-Pred: predicting the binding affinity of protein-DNA complexes using machine learning techniques and structural features. Methods 213: 10–17. 10.1016/j.ymeth.2023.03.002 36924867 PMC10563387

[RNA080646ALEC17] Harini K, Sekijima M, Gromiha MM. 2024. PRA-Pred: structure-based prediction of protein-RNA binding affinity. Int J Biol Macromol 259: 129490. 10.1016/j.ijbiomac.2024.129490 38224813

[RNA080646ALEC18] Hellman LM, Fried MG. 2007. Electrophoretic mobility shift assay (EMSA) for detecting protein–nucleic acid interactions. Nat Protoc 2: 1849–1861. 10.1038/nprot.2007.249 17703195 PMC2757439

[RNA080646ALEC19] Hess B, Bekker H, Berendsen HJC, Fraaije JGEM. 1997. LINCS: a linear constraint solver for molecular simulations. J Comput Chem 18: 1463–1472. 10.1002/(SICI)1096-987X(199709)18:12<1463::AID-JCC4>3.0.CO;2-H

[RNA080646ALEC20] Ho TK. 1995. Random decision forests. In Proceedings of 3rd International Conference on Document Analysis and Recognition, Vol. 1, pp. 278–282. IEEE.

[RNA080646ALEC21] Hong X, Tong X, Xie J, Liu P, Liu X, Song Q, Liu S, Liu S. 2023. An updated dataset and a structure-based prediction model for protein– RNA binding affinity. Proteins 91: 1245–1253. 10.1002/prot.26503 37186412

[RNA080646ALEC22] Jones S. 2016. Protein-RNA interactions: structural biology and computational modeling techniques. Biophys Rev 8: 359–367. 10.1007/s12551-016-0223-9 28510023 PMC5430296

[RNA080646ALEC23] Jorgensen WL, Chandrasekhar J, Madura JD, Impey RW, Klein ML. 1983. Refined TIP3P model for water. J Chem Phys 79: 926–935. 10.1063/1.445869

[RNA080646ALEC24] Katsamba P. 2002. Kinetic studies of RNA–protein interactions using surface plasmon resonance. Methods 26: 95–104. 10.1016/S1046-2023(02)00012-9 12054886

[RNA080646ALEC25] Kohavi R. 1995. A study of cross-validation and bootstrap for accuracy estimation and model selection. In Proceedings of the 14th International Joint Conference on Artificial Intelligence, Volume 2, IJCAI'95, pp. 1137–1143. Morgan Kaufmann Publishers Inc., San Francisco, CA.

[RNA080646ALEC26] Li Y, Shen J, Sun X, Li W, Liu G, Tang Y. 2010. Accuracy assessment of protein-based docking programs against RNA targets. J Chem Inf Model 50: 1134–1146. 10.1021/ci9004157 20481574

[RNA080646ALEC27] Lunde BM, Moore C, Varani G. 2007. RNA-binding proteins: modular design for efficient function. Nat Rev Mol Cell Biol 8: 479–490. 10.1038/nrm2178 17473849 PMC5507177

[RNA080646ALEC28] Nithin C, Mukherjee S, Bahadur RP. 2019. A structure-based model for the prediction of protein–RNA binding affinity. RNA 25: 1628–1645. 10.1261/rna.071779.119 31395671 PMC6859855

[RNA080646ALEC29] Pedregosa F, Michel V, Grisel O, Blondel M, Prettenhofer P, Weiss R, Vanderplas J, Cournapeau D, Pedregosa F, Varoquaux G, 2011. Scikit-learn: machine learning in Python. J Mach Learn Res 12: 2825–2830.

[RNA080646ALEC30] Pedretti A, Villa L, Vistoli G. 2004. VEGA–an open platform to develop chemo-bio-informatics applications, using plug-in architecture and script programming. J Comput Aided Mol Des 18: 167–173. 10.1023/B:JCAM.0000035186.90683.f2 15368917

[RNA080646ALEC31] Pettersen EF, Goddard TD, Huang CC, Couch GS, Greenblatt DM, Meng EC, Ferrin TE. 2004. UCSF Chimera—a visualization system for exploratory research and analysis. J Comput Chem 25: 1605–1612. 10.1002/jcc.20084 15264254

[RNA080646ALEC32] Pons C, Talavera D, De La Cruz X, Orozco M, Fernandez-Recio J. 2011. Scoring by intermolecular pairwise propensities of exposed residues (SIPPER): a new efficient potential for protein-protein docking. J Chem Inf Model 51: 370–377. 10.1021/ci100353e 21214199

[RNA080646ALEC33] Rio DC. 2012. Filter-binding assay for analysis of RNA–protein interactions. Cold Spring Harb Protoc 2012: pdb.prot071449. 10.1101/pdb.prot071449 23028069

[RNA080646ALEC34] Ryder SP, Recht MI, Williamson JR. 2008. Quantitative analysis of protein-RNA interactions by gel mobility shift. Methods Mol Biol 488: 99–115. 10.1007/978-1-60327-475-3_7 18982286 PMC2928675

[RNA080646ALEC35] Sievers F, Wilm A, Dineen D, Gibson TJ, Karplus K, Li W, Lopez R, McWilliam H, Remmert M, Söding J, 2011. Fast, scalable generation of high-quality protein multiple sequence alignments using Clustal Omega. Mol Syst Biol 7: 539. 10.1038/msb.2011.75 21988835 PMC3261699

[RNA080646ALEC36] Tian C, Kasavajhala K, Belfon KAA, Raguette L, Huang H, Migues AN, Bickel J, Wang Y, Pincay J, Wu Q, 2020. ff19SB: amino-acid-specific protein backbone parameters trained against quantum mechanics energy surfaces in solution. J Chem Theory Comput 16: 528–552. 10.1021/acs.jctc.9b00591 31714766 PMC13071887

[RNA080646ALEC37] Vivian JT, Callis PR. 2001. Mechanisms of tryptophan fluorescence shifts in proteins. Biophys J 80: 2093–2109. 10.1016/S0006-3495(01)76183-8 11325713 PMC1301402

[RNA080646ALEC38] Wang R, Fang X, Lu Y, Wang S. 2004. The PDBbind database: collection of binding affinities for protein-ligand complexes with known three-dimensional structures. J Med Chem 47: 2977–2980. 10.1021/jm030580l 15163179

[RNA080646ALEC39] Wolpert DH. 1992. Stacked generalization. Neural Netw 5: 241–259. 10.1016/S0893-6080(05)80023-1

[RNA080646ALEC40] Yang W, Deng L. 2020. PreDBA: a heterogeneous ensemble approach for predicting protein-DNA binding affinity. Sci Rep 10: 1278. 10.1038/s41598-020-57778-1 31992738 PMC6987227

[RNA080646ALEC41] Zar JH. 1972. Significance testing of the Spearman rank correlation coefficient. J Am Stat Assoc 67: 578–580. 10.1080/01621459.1972.10481251

[RNA080646ALEC42] Zgarbová M, Otyepka M, Šponer J, Mládek A, Banáš P, Cheatham TE, Jurečka P. 2011. Refinement of the Cornell et al. Nucleic acids force field based on reference quantum chemical calculations of glycosidic torsion profiles. J Chem Theory Comput 7: 2886–2902. 10.1021/ct200162x 21921995 PMC3171997

